# Differential gene expression signatures for cell wall integrity found in chitin synthase II *(chs2*Δ*) *and myosin II *(myo1*Δ*) *deficient cytokinesis mutants of *Saccharomyces cerevisiae*

**DOI:** 10.1186/1756-0500-2-87

**Published:** 2009-05-09

**Authors:** José F Rodríguez-Quiñones, José R Rodríguez-Medina

**Affiliations:** 1 Department of Biochemistry, School of Medicine, University of Puerto Rico, Medical Sciences Campus, San Juan, Puerto Rico

## Abstract

**Background:**

Myosin II-dependent contraction of the cytokinetic ring and primary septum formation by chitin synthase II are interdependent processes during cytokinesis in *Saccharomyces cerevisiae*. Hence, null mutants of myosin II *(myo1*Δ*) *and chitin synthase II *(chs2*Δ*) *share multiple morphological and molecular phenotypes. To understand the nature of their interdependent functions, we will seek to identify genes undergoing transcriptional regulation in *chs2*Δ strains and to establish a transcription signature profile for comparison with *myo1*Δ strains.

**Results:**

A total of 467 genes were commonly regulated between *myo1Δ *and *chs2Δ *mutant strains (p ≤ 0.01). Common regulated biological process categories identified by Gene Set Enrichment Analysis (GSEA) in both gene expression profiles were: protein biosynthesis, RNA processing, and stress response. Expression of 17/20 genes in the main transcriptional fingerprint for cell wall stress was confirmed in the *chs2Δ *strain versus 5/20 for the *myo1Δ *strain. One of these genes, *SLT2/MPK1*, was up-regulated in both strains and both strains accumulated the hyperphosphorylated form of Slt2p thereby confirming that the *PKC1 *cell wall integrity pathway (CWIP) was activated by both mutations. The *SLT2/MPK1 *gene, essential for *myo1Δ *strains, was not required in the *chs2Δ *strain.

**Conclusion:**

Comparison of the *chs2Δ *and *myo1*Δ gene expression profiles revealed similarities in the biological process categories that respond to the *chs2Δ *and *myo1*Δ gene mutations. This supports the view that these mutations affect a common function in cytokinesis. Despite their similarities, these mutants exhibited significant differences in expression of the main transcriptional fingerprint for cell wall stress and their requirement of the CWIP for survival.

## Background

In the budding yeast *Saccharomyces cerevisiae*, myosin type II (Myo1p) and chitin synthase II (Chs2p) are essential proteins for the formation of the normal cytokinetic apparatus. These proteins participate in assembly of the cytokinetic ring and synthesis of the primary septum, respectively, during cytokinesis [1-3]. Previous studies revealed that contraction of the cytokinetic ring and closure of the primary septum by Chs2p are interdependent processes that occur at adjacent sites on the plasma membrane and at similar times of the cell cycle [3]. Previous studies also showed that Chs2p at the bud neck is required to maintain the stability of the actomyosin ring and for normal completion of the cytokinetic process [2,4]. Thus, *MYO1 *and *CHS2 *deficient cells, (*myo1*Δ and *chs2*Δ respectively), have been described to be cytokinesis mutant strains [5] that share multiple phenotypes associated with their respective cytokinesis defect [3]. Both mutants require expression of chitin synthase III (Chs3p), [3,6,7] an enzyme that synthesizes ~90% of the cell wall chitin, which implies the existence of cell wall stress conditions. Cell wall stress can be overcome by activation of the *PKC1-*dependent cell wall integrity pathway [8,9]. Readouts of the pathway in yeast cells include up-regulation of cell wall biosynthetic enzymes, heat shock proteins, and increased production of other cell wall components [8].

In this study we will establish a signature transcription profile for *chs2*Δ strains and compare it with the signature profile in *myo1*Δ strains previously published [10]. To accomplish this goal, we have performed a comparative oligonucleotide microarray analysis of mRNAs extracted from a *chs2*Δ strain and wild-type controls. When compared to the profiles previously reported in *myo1*Δ strains, we observed that both mutants alter the expression of similar groups of genes associated with specific biological process categories. However, these mutants exhibit significant differences in expression of the main transcriptional fingerprint for cell wall stress and differ in their requirement of the cell wall integrity pathway for survival.

## Methods

### Strains and culture conditions

All the experiments were performed using *Saccharomyces cerevisiae *wild type, *chs2*Δ, and *myo1*Δstrains (Table 1). Cultures were grown overnight at 26°C between 0.5–0.8 OD_600 _in Complete Synthetic Medium (CSM)(2% glucose, 1× Nitrogen base), and CSM-HIS^- ^with continuous shaking at 200 rpm.

**Table 1:** Strains used in this study.

Strain	Genotype	Source
MGD353-46D (wild type)	MAT α *trp1–289 ura3–52 leu2–3, 112 his3delta1 *ADE^+ ^ARG *cyh*^*R*^	B. Rymond
YJR6 (*myo1*Δ)	MAT α *trp1–289 ura3–52 leu2–3, 112 his3delta1 *ADE^+ ^ARG *cyh*^*R *^*myo1delta::HIS5*^+ ^parental MGD353-46D	F. Rivera
YFR23 (*chs2*Δ*)*	MAT α *trp1–289 ura3–52 leu2–3, 112 his3delta1 *ADE^+ ^ARG *cyh*^*R*^*chs2delta::KAN*^*R *^parental MGD353-46D	F. Rivera
YJF2 (*chs2*Δ*slt2*Δ*)*	MAT α *trp1–289 ura3–52 leu2–3, 112 his3delta1 *ADE^+ ^ARG *cyh*^*R*^*chs2delta::KAN*^*R*^*slt2delta::URA3*^+ ^parental MGD353-46D	This study
YJF9 (*myo1*Δ*slt2*Δ pRS316-*MYO1)*	MAT α *trp1–289 ura3–52 leu2–3, 112 his3delta1 *ADE^+ ^ARG *cyh*^*R *^*myo1delta::HIS5*^+ ^parental MGD353-46D, pRS316-*MYO1*, *slt2delta::KAN*^*R*^	This study
YJF8 (*slt2*Δ*)*	MAT α *trp1–289 ura3–52 leu2–3, 112 his3delta1 *ADE^+ ^ARG *cyh*^*R*^, *slt2delta::KAN*^*R *^parental MGD353-46D	This study

### *SLT2 *gene disruption

A *SLT2 *gene disruption was created in the *myo1*Δ pRS316-*MYO1 *strain (Table 1) by replacing the *SLT2 *gene with a *KanMX4 *module by homologous recombination. The *myo1*Δ* slt2*Δ pRS316-*MYO1 *strain was grown in CSM 5-FOA to uncover the *myo1*Δ*slt2*Δ mutant [10]. Mutant *chs2*Δ strains were transformed with *URA3 *cassette, to perform the *SLT2 *gene disruption. All colonies were confirmed by PCR.

### RNA extraction procedure

Total RNA was extracted from cells derived from five biological replicate cultures of wild type and *chs2*Δ strains as described previously [10]. RNA concentrations, purity and integrity were determined using a Nanodrop spectrophotometer (Nanodrop Technologies, Wilmington, DE) and an Agilent Bioanalyzer (Agilent Technologies, Palo Alto, CA) respectively.

### Oligonucleotide microarray experiments

Oligonucleotide microarray experiments and data analysis were performed as described previously [10]. Briefly, 1.0 μg of total RNA from each sample was amplified using the Low RNA Input Fluorescent Linear Amplification kit (Agilent Technologies, Palo Alto, CA), and then it was labeled with 10 mM Cy3 or Cy5. Labeled cRNA's were hybridized, and then microarray slides were washed and scanned with a VersArray Chip Reader (BioRad, Hercules, CA). The microarrays raw data was generated with Imagene 3.0 and then analyzed using Limma software [11] as previously described [10]. The fold change in gene expression was calculated by 2^(M)^, where M is the log_2_-fold change after background correction and normalization. An Empirical Bayes Statistics [12]for differential expression analysis and FDR test [13] were performed. The p-value ≤ 0.01 cutoff was established for differential expression. Gene Set Enrichment Analysis (GSEA) [14] was performed using the Limma package of Bioconductor as described previously [10]. A corrected p-value was obtained from the analysis using the Bonferroni correction p-value ≤ 0.0004. Microarray raw and processed data are available at the Gene Expression Omnibus (GEO) site of NCBI (GSE5931 and GSE12994 for *myo1*Δ and *chs2*Δ, respectively) [15].

### Real time RT-PCR experiments

Real time RT-PCR assays were performed with 30 ng of total RNA using the Quantitec SYBR Green RT-PCR kit (Qiagen, Valencia, CA) as previously described [10]. The sequences of the forward and reverse primers for the selected mRNAs are listed in Table 2. The fold change was determined by the 2^ΔΔCt ^method [16] as described previously [10].

**Table 2:** Primers used in this study for real time RT-PCR and genetic deletions

Target	Forward primer	Reverse primer
*ACT1*	5'-GCCATTTTGAGAATCGATTTG-3'	5'-TTAGAAACACTTGTGGTGAAC-3'
*ECM4*	5'-GTGGTACAAACGGAGCTTTCA-3'	5'-GTGCCCAATGGACTACGCTACA-3'
*SPI1*	5'-CCAGAACCAACGACTTTCGTA-3'	5'-ACTGCACCAGCCAAACCTA-3'
*ROM1*	5'-AGCTATCTACGCCTCCAACT-3'	5'-ATGATGACGTTGGTGTTGA-3'
*SLT2*	5'-AGCAACAGCAGCCTTCAGA-3'	5'-GAACGCGAGGAAGTATCCAA-3'
*YHR097C*	5'-CCATCGTCGTACATCACAC-3'	5'-GTACAGGCGCCACTTTATTA-3'
*SLT2*pRS	5'-AATGGAAAGTTTCAGTGTTAAAAATAG AAACTGAAAAAGGAGATCTAGCCCGTTTCGGTGATGAC-3'	5'-AATAATGAATATTGTCTATAGATGACTAA TCATAAATGAACGAAAAGAAATTCCTGATGCGGTATTTTCTCC-3'
*ATG9*pRS	5'-TTACACTAATTAAGATGCTTAGATTCC CATTCAAAAGGTACTATTGACGTCGTTTCGGTGATGAC-3'	5'-GGCGGAAAGAAATAACCAATAATAA TAATTTATTAACCTCTTTTTTTCTTTTCCT GATGCGGTATTTTCTCCT-3'

### Western blot analysis of hyperphosphorylated Slt2p levels

Yeast strains were grown in selective medium between 0.5–0.8 OD_600 _at 26°C. Cells treatment, protein extraction and quantification methods were performed as described [6,9,10,17]. Total protein extracts (75 μg) were separated in a 10% SDS-PAGE gel and transferred to a nitrocellulose membrane at 70 V for 2 h at 4°C. The membrane was incubated with anti-phospho-p42/44 MAP kinase monoclonal antibody (1:1000) (Cell Signaling Technologies, Danvers, MA). The membrane was stripped and reprobed with a rabbit polyclonal antibody against Slt2p (1:1000) and mouse monoclonal antibody against Pgk1p (1:500) (Molecular probes, Invitrogen, Danvers, MA).

## Results and Discussion

### Comparison between transcriptional profiles of *chs2*Δ and *myo1*Δ strains

The rationale for these experiments was to identify differentially expressed genes and common biological process categories that are relevant to the myosin-dependent versus myosin-independent cytokinesis mechanisms operating in *chs2*Δ and *myo1*Δ strains respectively. Microarray hybridization experiments were conducted as described previously [10] with labeled total RNA obtained from five independent biological replicate cultures of the *chs2*Δ and wild type control strains (Table 1). A total of 467 genes were differentially expressed in common between *chs2*Δ and *myo1*Δ strains (p ≤ 0.01) and classified according to their biological processes [see Additional file 1]. The results of the oligonucleotide microarrays for the *chs2*Δ strain were validated by real time RT-PCR indicating a fold change of 1.11, 2.92, 7.21, 1.41, and 1.11 for *SLT2, ECM4, SPI1, YHR097C*, and *ROM1 *respectively that were consistent with the microarray results (Table 3).

**Table 3:** Confirmation of microarray data by real time RT-PCR assay on a selected set of genes for *chs2*Δ and *myo1*Δ[10] (p ≤ 0.01)

Gene name	Fold Change in Microarray *myo1*Δ[10]	Fold Change in Microarray *chs2*Δ	Fold Change (real time RT-PCR) *myo1*Δ[10]	Fold Change (real time RT-PCR) *chs2*Δ
*SLT2*	2.1	1.30	1.8	1.11
*ECM4*	3.2	2.13	3.5	2.92
*SPI1*	8.6	1.94	13.0	7.21
*YHR097C*	4.1	2.01	3.8	1.41
*ROM1*	3.2	1.30	3.5	1.11

**Figure 1:**
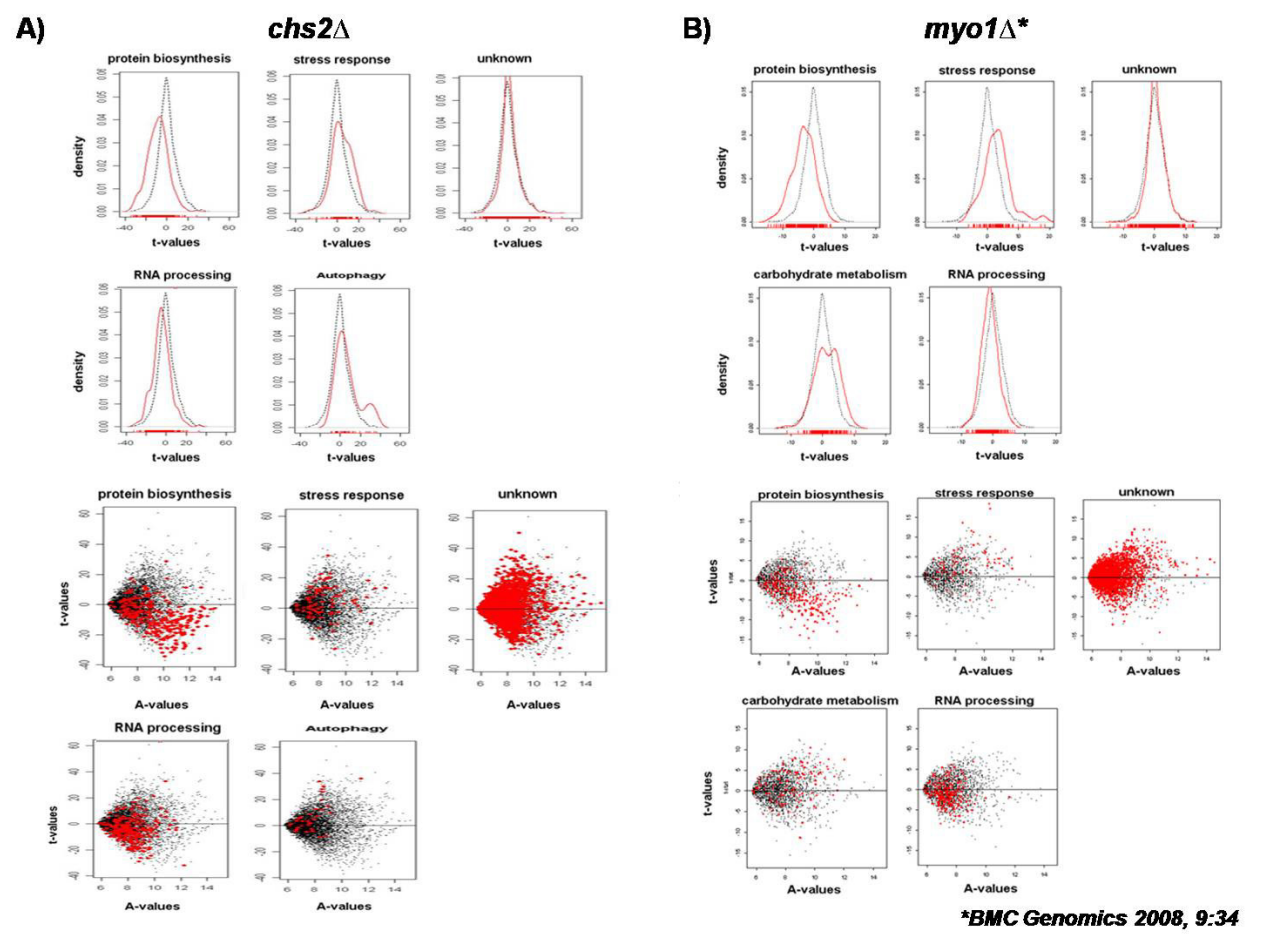
**Histograms derived from Gene Set Enrichment Analysis (GSEA) of *chs2*Δ and *myo1*Δ mutant strains**. The cutoff for significantly regulated biological process categories was based on a p-value ≤ 0.0004 as described in the Methods section. Red lines or dots represent the distribution of genes of the specific category in the array. Black lines or dots represent the distribution of genes for all categories in the array. (A, top panel) Density versus t-value plots generated for significant biological process categories from the *chs2*Δ strain. (A, bottom panel) t-value versus A-value plots for genes of the same biological process categories shown in (A, top panel). (B, top panel) Density versus t-value plots previously generated for significant biological process categories from the *myo1*Δ strain [10]. (B, bottom panel) t-value versus A-value plots for genes of the same biological process categories shown in (B, top panel). The results shown in Figure 1B were previously published in *BMC Genomics 2008, 9:34 *[10].

A comparison of results from GSEA of *chs2*Δ with those previously published for *myo1*Δ strains [10] revealed similar yet distinct transcription signature profiles. Five categories were identified with a corrected p-value below the cutoff (p ≤ 0.0004) for *chs2*Δ strains (Figure 1A, top and bottom panels). These categories were: protein biosynthesis, stress response, RNA processing, autophagy and genes encoding unknown biological processes. Histograms of density versus t-value were generated for each category to determine if regulation of a specific category occurred by comparing the distribution of genes in each biological process category relative to the normal distribution of all the categories represented on the array (Figure 1A, top panel). Plots of t-values versus A-values were created to identify the individual genes in each category and observe their distribution across the array (Figure 1A, bottom panel). Of the five biological process categories selected, the protein biosynthesis and RNA processing categories presented the most dramatic changes in their normal distribution (Figure 1A, top panel). The histogram reflecting the tendency of the protein biosynthesis category showed a shift from the normal distribution towards negative t-values. This was also observed in the corresponding t-value vs. A-value plot where we observe a greater quantity of genes biased towards negative t-values (Figure 1A, bottom panel). In the case of the RNA processing category, these genes were also shifted towards negative t-values (Figure 1A, top panel). These results are consistent with those obtained previously in *myo1*Δ mutant strains where the protein biosynthesis and RNA processing categories were biased towards negative t-values (Figure 1B top and bottom panels). Because levels of ribosome biogenesis and growth rate are correlated in budding yeast [18,19], down-regulation of protein biosynthesis genes is an indication that growth rate is also reduced, a phenotype that has been reported previously in both mutant strains [2,10]. Although the "unknown" biological process category had the greatest representation of differentially expressed genes in the array (670 genes), there was no shift observed in the density vs. t-values histogram or the t-values vs. A-values plot for *chs2*Δ (Figure 1A) suggesting that a similar numbers of genes were up and down regulated. The *myo1*Δ and *chs2*Δ mutant profiles differed in the autophagy and carbohydrate biological process categories.

### Analysis of the *PKC1*-dependent cell wall integrity pathway requirement

**Figure 2:**
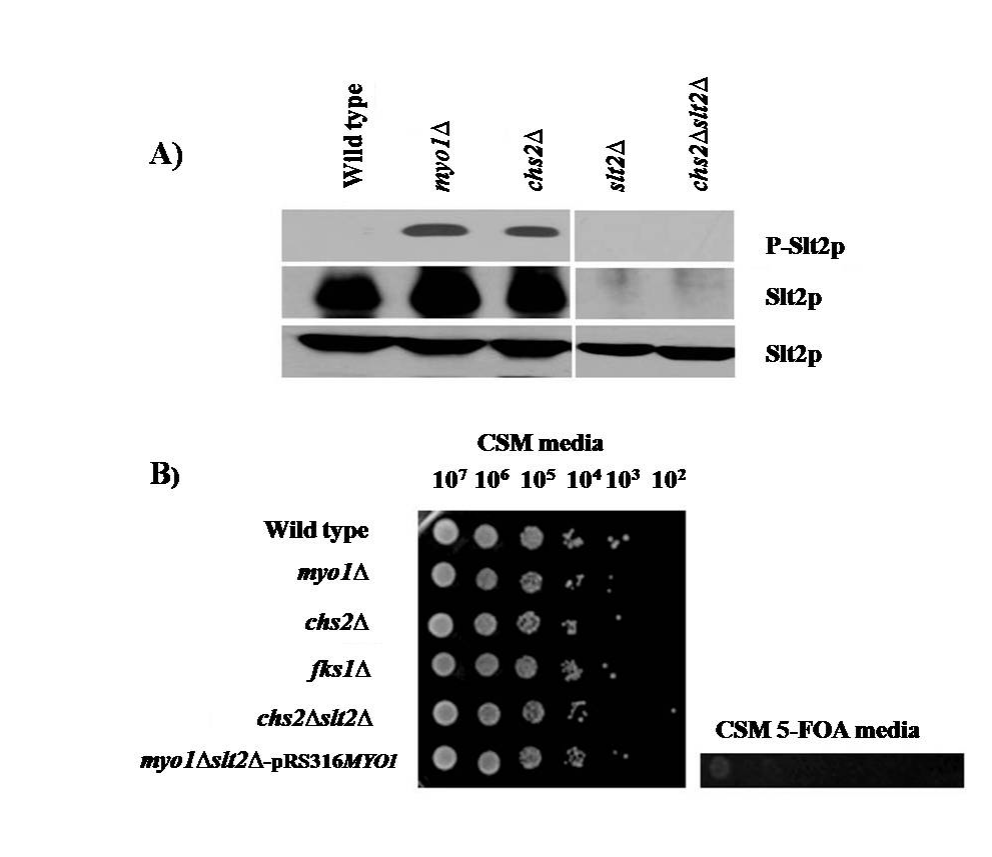
**Analysis of the *PKC1*-dependent cell wall integrity pathway in *chs2*Δ strains**. A) Western blot assay for activation of the *PKC1*-dependent cell wall integrity pathway in *chs2*Δ strains. Equal amounts of protein (75 μg) from wild type, *myo1*Δ, *chs2*Δ, *slt2*, and *chs2*Δ* slt2*Δ strains were analyzed by western blot to determine steady state levels of hyperphosphorylated Slt2p (p-Slt2p), total levels of Slt2p, and Pgk1p (a loading control) as described in the Methods section. B) Viability assays for wild type, *myo1*Δ, *chs2*Δ, *slt2*Δ, *chs2*Δ*slt2*Δ, and *myo1*Δ*slt2*ΔpRS316-*MYO1 *strains. Serial dilutions ranging from 1 × 10^7 ^– 1 × 10^2 ^cells/ml were plated on CSM media to observe growth after three days of culture at 29°C. The *myo1*Δ*slt2*Δ mutant was maintained by co-expressing a plasmid-borne copy of the *MYO1 *gene (pRS316-*MYO1*) as described previously [10]. Synthetic lethality was uncovered in this strain by negative selection for *URA3 *(contained on the plasmid) with 5-FOA.

A previous study of *myo1*Δ strains revealed that the *PKC1-*dependent pathway was activated by up-regulation of the *SLT2 *gene and an increase in hyperphosphorylated Slt2p (p-Slt2p) at steady state compared to their wild type [6,10,17]. In the *chs2*Δ gene expression profile, the *SLT2 *gene was also up-regulated (1.3-fold), and p-Slt2p levels were increased, thereby revealing *PKC1 *activation in this mutant (Figure 2A). However, the *slt2Δ *knockout mutation was viable in a *chs2Δ *strain although it was previously shown to be lethal in a *myo1*Δ strain (Figure 2B). The *slt2Δ *knockout mutation was confirmed in the *chs2Δ *strain by PCR (data not shown) and reconfirmed at the protein level by western blot (Figure 2A). These observations suggest that activation of the *PKC1*-dependent cell wall integrity pathway for survival is a *myo1Δ*-specific phenotype and may indicate that cell integrity defects are more severe in this strain.

A previous study described a transcriptional fingerprint consisting of 20 core genes that are a characteristic for cell wall stress caused by perturbation of cell wall integrity [20,21]. That study included the *fks1*Δ strain, which is considered a typical cell wall-deficient mutant and is characterized by the transcriptional up-regulation of the *SLT2 *gene and increase in the hyperphosphorylated form of Slt2p [20,21]. We performed confirmatory experiments in an *fks1*Δ strain and observed transcriptional activation of 17 of the described 20 core genes, thereby corroborating their results (data not shown). Despite the dispensability of Slt2p, the *chs2Δ *strain expressed 17 of the 20 core genes mentioned, in contrast to only 5 core genes activated in the *myo1*Δ strain (Table 4). Such a difference in transcription of these genes suggests that these two mutations affect cell wall integrity at different levels.

**Table 4:** Twenty genes representing the main transcriptional fingerprint for cell wall stress [20].

Gene name	*chs2*Δ	*myo1*Δ[10]
*YLR194C*	Up-regulated	
*YPL088W*		
*YIL023C*	Up-regulated	
*YLR414C*	Up-regulated	
*YHR097C*	Up-regulated	Up-regulated
*YAL053W*	Up-regulated	
*CWP1*	Up-regulated	
*SED1*	Up-regulated	Up-regulated
*PIR3*	Up-regulated	
*CRH1*	Up-regulated	
*KTR2*	Up-regulated	
*GFA1*	Up-regulated	
*PST1*	Up-regulated	
*SLT2*	Up-regulated	Up-regulated
*MLP1*	Up-regulated	
*YPS4*		
*FBP26*	Up-regulated	Up-regulated
*HSP12*	Up-regulated	Up-regulated
*PRM5*		
*SRL3*	Up-regulated	

Blank boxes signify no change in expression.

## Conclusion

In this study the global mRNA expression profile of a *chs2*Δ mutant strain was determined and analyzed by GSEA for further comparison with the previously reported *myo1*Δ strain profile [10]. The GSEA identified four biological categories affected by each mutant condition, where protein biosynthesis and RNA processing categories were down- regulated while stress response and autophagy were up-regulated. This analysis revealed that genes involved in the autophagy process were significantly up-regulated exclusively in the *chs2*Δ strain. We did not detect any growth impairment in an autophagy-deficient strain *chs2Δ atg9Δ *(data not shown) suggesting that although this represented a differentially regulated biological process in the *chs2*Δ strain, autophagy was not essential for growth of this mutant under our culture conditions.

We have demonstrated that the *PKC1-*dependent cell integrity pathway was activated in both *myo1Δ *and *chs2*Δ strains. Slt2p/Mpk1p, a gene product of the signal transduction biological process category, was not essential for cell viability in the *chs2Δ *mutant. Nonetheless, the *chs2*Δ strain exhibited higher resistance to cell lysis by exogenously added β-1, 3 glucanase than a wild-type strain suggesting that it most likely has modified its cell wall composition (data not shown). A positive contribution of the cell wall integrity pathway to these putative modifications may be inferred from the transcriptional fingerprint for this mutant.

In summary, a comparison of the *chs2Δ *and *myo1*Δ gene expression profiles revealed similarities in the biological process categories that respond to the *chs2Δ *and *myo1*Δ gene mutations. This supports the view that these mutations may affect common functions in cytokinesis. Despite their similarities, these mutants exhibited significant differences in expression of the main transcriptional fingerprint for cell wall stress and in their requirement of these genes for survival. These differences provide insight to how *S. cerevisiae *circumvents cell death and may help in the development of novel antifungal treatment.

## Competing interests

The authors declare that they have no competing interests.

## Authors' contributions

JFRQ participated in the microarray experiments and data analysis, real-time RT-PCR experiments, western blot experiments, and writing of the manuscript. JRRM participated as principal investigator in the design of experiments, analysis and interpretation of the data, and writing of the manuscript. All authors read and approved the final manuscript.

## Supplementary Material

Additional File 1 **Differentially expressed genes found in common between *myo1*Δ and *chs2*Δ strains (p ≤ 0.01).** Click here for file
