# Supplementary material for: Differential gene expression signatures for cell wall integrity found in chitin synthase II (chs2Δ) and myosin II (myo1Δ) deficient cytokinesis mutants of Saccharomyces cerevisiae
Source: BMC Res Notes. 2009 May 9;2:87. doi: 10.1186/1756-0500-2-87 (PMC2684100; doi:10.1186/1756-0500-2-87)
Supplement: Additional File 1 — Differentially expressed genes found in common between myo1Δ and chs2Δ strains (p ≤ 0.01). [file 1756-0500-2-87-S1.pdf]

YORF	Gene	Description	Fold change *myo1*Δ	Fold change *chs2*Δ
**Cell organization and biogenesis**				
YDL014W	*NOP1*	Nop1p|Nucleolar protein, component of the small subunit processome complex, which is required for processing of pre-18S rRNA; has similarity to mammalian fibrillarin; protein coding	-2.62	-2.04
YOL077C	*BRX1*	Brx1p|Nucleolar protein, constituent of 66S pre-ribosomal particles; depletion leads to defects in rRNA processing and a block in the assembly of large ribosomal subunits; possesses a sigma(70)-like RNA-binding motif; protein coding	-2.08	-2.76
YDR465C	*RMT2*	Arginine methyltransferase; ribosomal protein L12 is a substrate|Rmt2p; protein coding	-2.40	-3.86
YPL226W	*NEW1*	ATP binding cassette family member; Asn/Gln-rich rich region supports [NU+] prion formation, susceptibility to [PSI+] prion induction and aggregation of a fragment of the human Machado-Joseph Disease protein|New1p; protein coding	-2.34	-2.57
YKR076W	*ECM4*	Ecm4p|Omega class glutathione transferase; not essential; similar to Ygr154cp; green fluorescent protein (GFP)-fusion protein localizes to the cytoplasm; protein coding	3.26	2.13
YKR057W	*RPS21A*	Protein component of the small (40S) ribosomal subunit; nearly identical to Rps21Bp and has similarity to rat S21 ribosomal protein|Rps21ap; protein coding	-2.32	-1.98
YDR418W	*RPL12B*	Protein component of the large (60S) ribosomal subunit, nearly identical to Rpl12Ap; rpl12a rpl12b double mutant exhibits slow growth and slow translation; has similarity to E. coli L11 and rat L12 ribosomal	-3.28	-2.92

		proteins|Rpl12bp; protein coding		
YOL004W	*SIN3*	Component of the Sin3p-Rpd3p histone deacetylase complex, involved in transcriptional repression and activation of diverse processes, including mating-type switching and meiosis; involved in the maintenance of chromosomal integrity|Sin3p; protein coding	2.29	-1.13
YCL059C	*KRR1*	Essential nucleolar protein required for the synthesis of 18S rRNA and for the assembly of 40S ribosomal subunit|Krr1p; protein coding	-2.37	-1.80
YIL052C	*RPL34B*	Protein component of the large (60S) ribosomal subunit, nearly identical to Rpl34Ap and has similarity to rat L34 ribosomal protein|Rpl34bp; protein coding	-2.13	-1.63
YGL135W	*RPL1B*	N-terminally acetylated protein component of the large (60S) ribosomal subunit, nearly identical to Rpl1Ap and has similarity to E. coli L1 and rat L10a ribosomal proteins; rpl1a rpl1b double null mutation is lethal|Rpl1bp; protein coding	-2.65	-4.96
YGL120C	*PRP43*	Prp43p|RNA helicase in the DEAH-box family, functions in both RNA polymerase I and polymerase II transcript metabolism, involved in release of the lariat-intron from the spliceosome; protein coding	-2.33	1.47
YNL312W	*RFA2*	Rfa2p|Subunit of heterotrimeric Replication Protein A (RPA), which is a highly conserved single-stranded DNA binding protein involved in DNA replication, repair, and recombination; protein coding	-2.00	-1.52
YGL099W	*LSG1*	Lsg1p|Putative GTPase involved in 60S ribosomal subunit biogenesis; required for the release of Nmd3p from 60S subunits in the cytoplasm; protein coding	-1.95	-1.31
YML034W	*SRC1*	Inner nuclear membrane (INM) protein with a putative role in sister chromatid segregation, potentially phosphorylated by Cdc28p; contains	2.17	1.25

		helix-extension-helix (HEH) motif, nuclear localization signal sequence|Src1p; protein coding		
YDR309C	*GIC2*	Gic2p|Protein of unknown function involved in initiation of budding and cellular polarization, interacts with Cdc42p via the Cdc42/Rac-interactive binding (CRIB) domain; protein coding	-2.40	2.17
YDR300C	*PRO1*	Gamma-glutamyl kinase, catalyzes the first step in proline biosynthesis|Pro1p; protein coding	-1.45	-2.8
YNL283C	*WSC2*	Partially redundant sensor-transducer of the stress-activated PKC1-MPK1 signaling pathway involved in maintenance of cell wall integrity and recovery from heat shock; secretory pathway Wsc2p is required for the arrest of secretion response|Wsc2p; protein coding	-1.96	1.48
YHR203C	*RPS4B*	Protein component of the small (40S) ribosomal subunit; identical to Rps4Ap and has similarity to rat S4 ribosomal protein|Rps4bp; protein coding	-2.67	-2.90
YKL113C	*RAD27*	5' to 3' exonuclease, 5' flap endonuclease, required for Okazaki fragment processing and maturation as well as for long-patch base-excision repair; member of the S. pombe RAD2/FEN1 family|Rad27p; protein coding	-1.89	-1.04
YHR170W	*NMD3*	Nmd3p|Protein involved in nuclear export of the large ribosomal subunit; acts as a Crm1p-dependent adapter protein for export of nascent ribosomal subunits through the nuclear pore complex; protein coding	-2.01	-3.09
YDR225W	*HTA1*	Hta1p|One of two nearly identical (see also HTA2) histone H2A subtypes; core histone required for chromatin assembly and chromosome function; DNA damage-dependent phosphorylation by Mec1p facilitates DNA repair; acetylated by Nat4p; protein coding	-5.92	-1.55

YDR224C	*HTB1*	Htb1p|One of two nearly identical (see HTB2) histone H2B subtypes required for chromatin assembly and chromosome function; Rad6p-Bre1p-Lge1p mediated ubiquitination regulates transcriptional activation, meiotic DSB formation and H3 methylation; protein coding	-2.36	1.69
YHR139C	*SPS100*	Protein required for spore wall maturation; expressed during sporulation; may be a component of the spore wall|Sps100p; protein coding	6.98	2.50
YLR409C	*UTP21*	Possible U3 snoRNP protein involved in maturation of pre-18S rRNA, based on computational analysis of large-scale protein-protein interaction data|Utp21p; protein coding	-2.04	-2.24
YOR340C	*RPA43*	RNA polymerase I subunit A43|Rpa43p; protein coding	-2.06	-1.26
YKL013C	*ARC19*	Arc19p|Subunit of the ARP2/3 complex, which is required for the motility and integrity of cortical actin patches; protein coding	-1.53	-1.20
YKL009W	*MRT4*	Mrt4p|Protein involved in mRNA turnover and ribosome assembly, localizes to the nucleolus; protein coding	-3.61	-3.29
YOR312C	*RPL20B*	Protein component of the large (60S) ribosomal subunit, nearly identical to Rpl20Ap and has similarity to rat L18a ribosomal protein|Rpl20bp; protein coding	-3.33	-2.53
YJR145C	*RPS4A*	Protein component of the small (40S) ribosomal subunit; mutation affects 20S pre-rRNA processing; identical to Rps4Bp and has similarity to rat S4 ribosomal protein|Rps4ap; protein coding	-2.79	-2.70
YDR129C	*SAC6*	Fimbrin, actin-bundling protein; cooperates with Scp1p (calponin/transgelin) in the organization and maintenance of the actin cytoskeleton|Sac6p; protein coding	1.92	-1.04

YBR106W	*PHO88*	Pho88p|Probable membrane protein, involved in phosphate transport; pho88 pho86 double null mutant exhibits enhanced synthesis of repressible acid phosphatase at high inorganic phosphate concentrations; protein coding	-2.76	-2.30
YHR030C	*SLT2*	Serine/threonine MAP kinase involved in regulating the maintenance of cell wall integrity and progression through the cell cycle; regulated by the PKC1-mediated signaling pathway|Slt2p; protein coding	1.72	1.29
YBR084W	*MIS1*	Mis1p|Mitochondrial C1-tetrahydrofolate synthase, involved in interconversion between different oxidation states of tetrahydrofolate (THF); provides activities of formyl-THF synthetase, methenyl-THF cyclohydrolase, and methylene-THF dehydrogenase; protein coding	-2.04	-1.91
YOR241W	*MET7*	Folylpolyglutamate synthetase, catalyzes extension of the glutamate chains of the folate coenzymes, required for methionine synthesis and for maintenance of mitochondrial DNA|Met7p; protein coding	-1.98	-1.82
YNL066W	*SUN4*	Cell wall protein related to glucanases, possibly involved in cell wall septation; member of the SUN family|Sun4p; protein coding	-6.61	-1.06
YBR048W	*RPS11B*	Protein component of the small (40S) ribosomal subunit; identical to Rps11Ap and has similarity to E. coli S17 and rat S11 ribosomal proteins|Rps11bp; protein coding	-4.04	-3.27
YHL033C	*RPL8A*	Ribosomal protein L4 of the large (60S) ribosomal subunit, nearly identical to Rpl8Bp and has similarity to rat L7a ribosomal protein; mutation results in decreased amounts of free 60S subunits|Rpl8ap; protein coding	-3.16	-3.34
YDR060W	*MAK21*	Constituent of 66S pre-ribosomal particles, required for large (60S)	-2.30	-2.99

		ribosomal subunit biogenesis; involved in nuclear export of pre-ribosomes; required for maintenance of dsRNA virus; homolog of human CAATT-binding protein|Mak21p; protein coding		
YLR249W	*YEF3*	Translational elongation factor 3, stimulates the binding of aminoacyl-tRNA (AA-tRNA) to ribosomes by releasing EF-1 alpha from the ribosomal complex; contains two ABC cassettes; binds and hydrolyses ATP|Yef3p; protein coding	-2.16	-1.84
YOR198C	*BFR1*	Bfr1p|Component of mRNP complexes associated with polyribosomes; implicated in secretion and nuclear segregation; multicopy suppressor of BFA (Brefeldin A) sensitivity; protein coding	-2.38	1.26
YJR043C	*POL32*	Pol32p|Third subunit of DNA polymerase delta, involved in chromosomal DNA replication; required for error-prone DNA synthesis in the presence of DNA damage and processivity; interacts with Hys2p, PCNA (Pol30p), and Pol1p; protein coding	-1.74	-1.22
YER116C	*SLX8*	Slx8p|Subunit of the Slx5-Slx8 substrate-specific ubiquitin ligase complex; stimulated by prior attachment of SUMO to the substrate; protein coding	1.85	2.01
YLR197W	*SIK1*	Essential evolutionarily-conserved nucleolar protein component of the box C/D snoRNP complexes that direct 2'-O-methylation of pre-rRNA during its maturation; overexpression causes spindle orientation defects|Sik1p; protein coding	-2.55	-1.20
YMR307W	*GAS1*	Beta-1,3-glucanosyltransferase, required for cell wall assembly; localizes to the cell surface via a glycosylphosphatidylinositol (GPI) anchor|Gas1p; protein coding	-2.87	1.44

YMR302C	*PRP12*	Integral inner mitochondrial membrane protein with a role in maintaining mitochondrial nucleoid structure and number; mutants exhibit an increased rate of mitochondrial DNA escape; shows some sequence similarity to exonucleases|Yme2p; protein coding	2.13	1.23
YLR167W	*RPS31*	Fusion protein that is cleaved to yield a ribosomal protein of the small (40S) subunit and ubiquitin; ubiquitin may facilitate assembly of the ribosomal protein into ribosomes; interacts genetically with translation factor eIF2B|Rps31p; protein coding	-2.48	-5.21
YDL226C	*GCS1*	ADP-ribosylation factor GTPase activating protein (ARF GAP), involved in ER-Golgi transport; shares functional similarity with Glo3p|Gcs1p; protein coding	-1.98	-1.54
YJL190C	*RPS22A*	Protein component of the small (40S) ribosomal subunit; nearly identical to Rps22Bp and has similarity to E. coli S8 and rat S15a ribosomal proteins|Rps22ap; protein coding	-3.24	-4.07
YER070W	*RNR1*	Ribonucleotide-diphosphate reductase (RNR), large subunit; the RNR complex catalyzes the rate-limiting step in dNTP synthesis and is regulated by DNA replication and DNA damage checkpoint pathways via localization of the small subunits|Rnr1p; protein coding	-4.62	-2.13
YDL198C	*GGC1*	Ggc1p|Mitochondrial GTP/GDP transporter, essential for mitochondrial genome maintenance; has a role in mitochondrial iron transport; member of the mitochondrial carrier family; protein coding	3.35	3.93
YDL192W	*ARF1*	ADP-ribosylation factor, GTPase of the Ras superfamily involved in regulation of coated vesicle formation in intracellular trafficking within the Golgi; functionally interchangeable with Arf2p|Arf1p; protein coding	-2.00	-1.53

YBL035C	*POL12*	B subunit of DNA polymerase alpha-primase complex, required for initiation of DNA replication during mitotic and premeiotic DNA synthesis; also functions in telomere capping and length regulation|Pol12p; protein coding	-2.12	-1.53
YDL188C	*PPH22*	Catalytic subunit of protein phosphatase 2A, functionally redundant with Pph21p; methylated at C terminus; forms alternate complexes with several regulatory subunits; involved in signal transduction and regulation of mitosis|Pph22p; protein coding	1.68	-1.07
YBL032W	*HEK2*	Hek2p|RNA binding protein with similarity to hnRNP-K that localizes to the cytoplasm and to subtelomeric DNA; required for the proper localization of ASH1 mRNA; involved in the regulation of telomere position effect and telomere length; protein coding	-1.71	1.40
YMR241W	*YHM2*	Mitochondrial DNA-binding protein, component of the mitochondrial nucleoid structure, involved in mtDNA replication and segregation of mitochondrial genomes; member of the mitochondrial carrier protein family|Yhm2p; protein coding	-2.69	-1.88
YBL003C	*HTA2*	Hta2p|One of two nearly identical (see also HTA1) histone H2A subtypes; core histone required for chromatin assembly and chromosome function; DNA damage-dependent phosphorylation by Mec1p facilitates DNA repair; acetylated by Nat4p; protein coding	-6.53	-1.57
YLR090W	*XDJ1*	Putative chaperone, homolog of E. coli DnaJ, closely related to Ydj1p; the authentic, non-tagged protein is detected in highly purified mitochondria in high-throughput studies|Xdj1p; protein coding	1.59	1.04
YER006W	*NUG1*	GTPase that associates with nuclear 60S pre-ribosomes, required for	-1.93	-3.21

		export of 60S ribosomal subunits from the nucleus|Nug1p; protein coding		
YDL143W	*CCT4*	Cct4p|Subunit of the cytosolic chaperonin Cct ring complex, related to Tcp1p, required for the assembly of actin and tubulins in vivo; protein coding	-1.50	-1.79
YMR202W	*ERG2*	C-8 sterol isomerase, catalyzes the isomerization of the delta-8 double bond to the delta-7 position at an intermediate step in ergosterol biosynthesis|Erg2p; protein coding	-4.03	-1.47
YGR123C	*PPT1*	Ppt1p|Protein serine/threonine phosphatase with similarity to human phosphatase PP5; present in both the nucleus and cytoplasm; expressed during logarithmic growth; computational analyses suggest roles in phosphate metabolism and rRNA processing; protein coding	-1.91	-3.29
YJL074C	*SMC3*	Smc3p|Subunit of the multiprotein cohesin complex required for sister chromatid cohesion in mitotic cells; also required, with Rec8p, for cohesion and recombination during meiosis; phylogenetically conserved SMC chromosomal ATPase family member; protein coding	-2.02	-1.78
YDL083C	*RPS16B*	Protein component of the small (40S) ribosomal subunit; identical to Rps16Ap and has similarity to E. coli S9 and rat S16 ribosomal proteins|Rps16bp; protein coding	-3.23	-3.08
YMR142C	*RPL13B*	Protein component of the large (60S) ribosomal subunit, nearly identical to Rpl13Ap; not essential for viability; has similarity to rat L13 ribosomal protein|Rpl13bp; protein coding	-2.16	-2.19
YAL025C	*MAK16*	Essential nuclear protein, constituent of 66S pre-ribosomal particles; required for maturation of 25S and 5.8S rRNAs; required for maintenance of M1 satellite double-stranded RNA of the L-A virus|Mak16p; protein coding	-3.03	-1.97

YJL042W	*MHP1*	Mhp1p|Microtubule-associated protein involved in assembly and stabilization of microtubules; overproduction results in cell cycle arrest at G2 phase; similar to Drosophila protein MAP and to mammalian MAP4 proteins; protein coding	2.29	1.29
YGR070W	*ROM1*	GDP/GTP exchange protein (GEP) for Rho1p; mutations are synthetically lethal with mutations in rom2, which also encodes a GEP|Rom1p; protein coding	2.81	1.34
YOL121C	*RPS19A*	Protein component of the small (40S) ribosomal subunit, required for assembly and maturation of pre-40 S particles; mutations in human RPS19 are associated with Diamond Blackfan anemia; nearly identical to Rps19Bp|Rps19ap; protein coding	-2.12	-2.18
YMR116C	*ASC1*	Asc1p|G-protein beta subunit and guanine nucleotide dissociation inhibitor for Gpa2p; ortholog of RACK1 that inhibits translation; core component of the small (40S) ribosomal subunit; represses Gcn4p in the absence of amino acid starvation; ncRNA	-3.77	-3.05
YLL045C	*RPL8B*	Ribosomal protein L4 of the large (60S) ribosomal subunit, nearly identical to Rpl8Ap and has similarity to rat L7a ribosomal protein; mutation results in decreased amounts of free 60S subunits|Rpl8bp; protein coding	-2.55	-4.72
**YORF**	**Gene**	**Description**	**Fold change *myo1*Δ**	**Fold change *chs2*Δ**
**Protein biosynthesis**				
YGR034W	*RPL26B*	Protein component of the large (60S) ribosomal subunit, nearly identical to Rpl26Ap and has similarity to E. coli L24 and rat L26 ribosomal proteins;	-2.60	-1.99

		binds to 5.8S rRNA|Rpl26bp; protein coding		
YPL249C-A	*RPL36B*	Protein component of the large (60S) ribosomal subunit, nearly identical to Rpl36Ap and has similarity to rat L36 ribosomal protein; binds to 5.8 S rRNA|Rpl36bp; protein coding	-2.29	-1.89
YDR471W	*RPL27B*	Protein component of the large (60S) ribosomal subunit, nearly identical to Rpl27Ap and has similarity to rat L27 ribosomal protein|Rpl27bp; protein coding	-2.55	-1.80
YPL220W	*RPL1A*	N-terminally acetylated protein component of the large (60S) ribosomal subunit, nearly identical to Rpl1Bp and has similarity to E. coli L1 and rat L10a ribosomal proteins; rpl1a rpl1b double null mutation is lethal|Rpl1ap; protein coding	-2.48	-4.36
YCR046C	*IMG1*	Img1p|Mitochondrial ribosomal protein of the large subunit, required for respiration and for maintenance of the mitochondrial genome; protein coding	-1.58	-1.25
YIL133C	*RPL16A*	N-terminally acetylated protein component of the large (60S) ribosomal subunit, binds to 5.8 S rRNA; has similarity to Rpl16Bp, E. coli L13 and rat L13a ribosomal proteins; transcriptionally regulated by Rap1p|Rpl16ap; protein coding	-2.71	-2.46
YGL189C	*RPS26A*	Protein component of the small (40S) ribosomal subunit; nearly identical to Rps26Bp and has similarity to rat S26 ribosomal protein|Rps26ap; protein coding	-2.12	-2.62
YPL143W	*RPL33A*	N-terminally acetylated ribosomal protein L37 of the large (60S) ribosomal subunit, nearly identical to Rpl33Bp and has similarity to rat L35a; rpl33a null mutant exhibits slow growth while rpl33a rpl33b double null mutant is inviable|Rpl33ap; protein coding	-1.86	-1.54
YPL131W	*RPL5*	Protein component of the large (60S) ribosomal subunit with similarity to	-2.40	-3.71

		E. coli L18 and rat L5 ribosomal proteins; binds 5S rRNA and is required for 60S subunit assembly|Rpl5p; protein coding		
YGL147C	*RPL9A*	Protein component of the large (60S) ribosomal subunit, nearly identical to Rpl9Bp and has similarity to E. coli L6 and rat L9 ribosomal proteins|Rpl9ap; protein coding	-2.39	-4.36
YML073C	*RPL6A*	N-terminally acetylated protein component of the large (60S) ribosomal subunit, has similarity to Rpl6Bp and to rat L6 ribosomal protein; binds to 5.8S rRNA|Rpl6ap; protein coding	-2.51	-2.32
YML063W	*RPS1B*	Ribosomal protein 10 (rp10) of the small (40S) subunit; nearly identical to Rps1Ap and has similarity to rat S3a ribosomal protein|Rps1bp; protein coding	-3.28	-2.41
YKL180W	*RPL17A*	Protein component of the large (60S) ribosomal subunit, nearly identical to Rpl17Bp and has similarity to E. coli L22 and rat L17 ribosomal proteins; copurifies with the Dam1 complex (aka DASH complex)|Rpl17ap; protein coding	-3.49	-2.46
YGL123W	*RPS2*	Protein component of the small (40S) subunit, essential for control of translational accuracy; has similarity to E. coli S5 and rat S2 ribosomal proteins|Rps2p; protein coding	-2.40	-4.09
YIL018W	*RPL2B*	Protein component of the large (60S) ribosomal subunit, identical to Rpl2Ap and has similarity to E. coli L2 and rat L8 ribosomal proteins; expression is upregulated at low temperatures|Rpl2bp; protein coding	-3.24	-1.89
YPL090C	*RPS6A*	Protein component of the small (40S) ribosomal subunit; identical to Rps6Bp and has similarity to rat S6 ribosomal protein|Rps6ap; protein coding	-2.52	-2.10
YPL079W	*RPL21B*	Protein component of the large (60S) ribosomal subunit, nearly identical to Rpl21Ap and has similarity to rat L21	-3.22	-3.09

		ribosomal protein|Rpl21bp; protein coding		
YGL076C	*RPL7A*	Protein component of the large (60S) ribosomal subunit, nearly identical to Rpl7Bp and has similarity to E. coli L30 and rat L7 ribosomal proteins; contains a conserved C-terminal Nucleic acid Binding Domain (NDB2)|Rpl7ap; ncRNA	-1.82	-1.51
YGL031C	*RPL24A*	Ribosomal protein L30 of the large (60S) ribosomal subunit, nearly identical to Rpl24Bp and has similarity to rat L24 ribosomal protein; not essential for translation but may be required for normal translation rate|Rpl24ap; protein coding	-2.67	-1.51
YGL030W	*RPL30*	Protein component of the large (60S) ribosomal subunit, has similarity to rat L30 ribosomal protein; involved in pre-rRNA processing in the nucleolus; autoregulates splicing of its transcript|Rpl30p; protein coding	-3.01	-1.77
YKL081W	*TEF4*	Tef4p|Translation elongation factor EF-1 gamma; ncRNA	-2.90	-1.15
YBR191W	*RPL21A*	Protein component of the large (60S) ribosomal subunit, nearly identical to Rpl21Bp and has similarity to rat L21 ribosomal protein|Rpl21ap; protein coding	-3.75	-2.78
YBR189W	*RPS9B*	Protein component of the small (40S) ribosomal subunit; nearly identical to Rps9Ap and has similarity to E. coli S4 and rat S9 ribosomal proteins|Rps9bp; protein coding	-2.28	-3.97
YBR181C	*RPS6B*	Protein component of the small (40S) ribosomal subunit; identical to Rps6Ap and has similarity to rat S6 ribosomal protein|Rps6bp; protein coding	-2.51	-2.34
YLR388W	*RPS29A*	Protein component of the small (40S) ribosomal subunit; nearly identical to Rps29Bp and has similarity to rat S29 and E. coli S14 ribosomal proteins|Rps29ap; protein coding	-2.01	-1.82

YFR032C-A	*RPL29*	Protein component of the large (60S) ribosomal subunit, has similarity to rat L29 ribosomal protein; not essential for translation, but required for proper joining of the large and small ribosomal subunits and for normal translation rate|Rpl29p; protein coding	-1.88	-2.14
YFR031C-A	*RPL2A*	Protein component of the large (60S) ribosomal subunit, identical to Rpl2Bp and has similarity to E. coli L2 and rat L8 ribosomal proteins|Rpl2ap; protein coding	-3.62	-1.52
YNL162W	*RPL42A*	Protein component of the large (60S) ribosomal subunit, identical to Rpl42Bp and has similarity to rat L44 ribosomal protein|Rpl42ap; protein coding	-2.43	-3.06
YLR344W	*RPL26A*	Protein component of the large (60S) ribosomal subunit, nearly identical to Rpl26Bp and has similarity to E. coli L24 and rat L26 ribosomal proteins; binds to 5.8S rRNA|Rpl26ap; protein coding	-2.56	-1.83
YBR121C	*GRS1*	Cytoplasmic and mitochondrial glycyl-tRNA synthase that ligates glycine to the cognate anticodon bearing tRNA; transcription termination factor that may interact with the 3'-end of pre-mRNA to promote 3'-end formation|Grs1p; protein coding	-2.08	-2.43
YFL022C	*FRS2*	Alpha subunit of cytoplasmic phenylalanyl-tRNA synthetase, forms a tetramer with Frs1p to form active enzyme; evolutionarily distant from mitochondrial phenylalanyl-tRNA synthetase based on protein sequence, but substrate binding is similar|Frs2p; protein coding	-1.81	-1.75
YNL096C	*RPS7B*	Protein component of the small (40S) ribosomal subunit, nearly identical to Rps7Ap; interacts with Kti11p; deletion causes hypersensitivity to zymocin; has similarity to rat S7 and Xenopus S8 ribosomal	-2.13	-1.16

		proteins|Rps7bp; protein coding		
YNL069C	*RPL16B*	N-terminally acetylated protein component of the large (60S) ribosomal subunit, binds to 5.8 S rRNA; has similarity to Rpl16Ap, E. coli L13 and rat L13a ribosomal proteins; transcriptionally regulated by Rap1p|Rpl16bp; protein coding	-1.84	-2.64
YNL067W	*RPL9B*	Protein component of the large (60S) ribosomal subunit, nearly identical to Rpl9Ap and has similarity to E. coli L6 and rat L9 ribosomal proteins|Rpl9bp; protein coding	-2.38	-3.19
YER131W	*RPS26B*	Protein component of the small (40S) ribosomal subunit; nearly identical to Rps26Ap and has similarity to rat S26 ribosomal protein|Rps26bp; protein coding	-2.76	-3.55
YDR025W	*RPS11A*	Protein component of the small (40S) ribosomal subunit; identical to Rps11Bp and has similarity to E. coli S17 and rat S11 ribosomal proteins|Rps11ap; protein coding	-3.19	-2.92
YER102W	*RPS8B*	Protein component of the small (40S) ribosomal subunit; identical to Rps8Ap and has similarity to rat S8 ribosomal protein|Rps8bp; protein coding	-2.53	-2.97
YGR264C	*MES1*	Mes1p|Methionyl-tRNA synthetase, forms a complex with glutamyl-tRNA synthetase (Gus1p) and Arc1p, which increases the catalytic efficiency of both tRNA synthetases; also has a role in nuclear export of tRNAs; protein coding	-2.04	-2.97
YER074W	*RPS24A*	Protein component of the small (40S) ribosomal subunit; identical to Rps24Bp and has similarity to rat S24 ribosomal protein|Rps24ap; protein coding	-2.06	-2.29
YJL177W	*RPL17B*	Protein component of the large (60S) ribosomal subunit, nearly identical to Rpl17Ap and has similarity to E. coli L22 and rat L17 ribosomal proteins|Rpl17bp; protein coding	-4.81	-2.01

YGR214W	*RPS0A*	Protein component of the small (40S) ribosomal subunit, nearly identical to Rps0Bp; required for maturation of 18S rRNA along with Rps0Bp; deletion of either RPS0 gene reduces growth rate, deletion of both genes is lethal|Rps0ap; protein coding	-2.67	-1.94
YER056C-A	*RPL34A*	Protein component of the large (60S) ribosomal subunit, nearly identical to Rpl34Bp and has similarity to rat L34 ribosomal protein|Rpl34ap; protein coding	-2.96	-2.43
YOR096W	*RPS7A*	Protein component of the small (40S) ribosomal subunit, nearly identical to Rps7Bp; interacts with Kti11p; deletion causes hypersensitivity to zymocin; has similarity to rat S7 and Xenopus S8 ribosomal proteins|Rps7ap; protein coding	-2.62	-2.49
YDL184C	*RPL41A*	Ribosomal protein L47 of the large (60S) ribosomal subunit, identical to Rpl41Bp and has similarity to rat L41 ribosomal protein; comprised of only 25 amino acids; rpl41a rpl41b double null mutant is viable|Rpl41ap; protein coding	-1.99	-3.33
YGR185C	*TYS1*	Cytoplasmic tyrosyl-tRNA synthetase, class I aminoacyl-tRNA synthetase that aminoacylates tRNA(Tyr), required for cytoplasmic protein synthesis, interacts with positions 34 and 35 of the anticodon of tRNATyr|Tys1p; protein coding	-1.64	-1.68
YDL136W	*RPL35B*	Protein component of the large (60S) ribosomal subunit, identical to Rpl35Ap and has similarity to rat L35 ribosomal protein|Rpl35bp; protein coding	-2.21	-1.89
YDL133C-A	*RPL41B*	Ribosomal protein L47 of the large (60S) ribosomal subunit, identical to Rpl41Ap and has similarity to rat L41 ribosomal protein; comprised of only 25 amino acids; rpl41a rpl41b double null mutant is viable|Rpl41bp; protein coding	-2.13	-3.45

YPR102C	*RPL11A*	Protein component of the large (60S) ribosomal subunit, nearly identical to Rpl11Bp; involved in ribosomal assembly; depletion causes degradation of proteins and RNA of the 60S subunit; has similarity to E. coli L5 and rat L11|Rpl11ap; protein coding	-2.77	-2.80
YLR061W	*RPL22A*	Protein component of the large (60S) ribosomal subunit, has similarity to Rpl22Bp and to rat L22 ribosomal protein|Rpl22ap; protein coding	-3.89	-2.86
YLR060W	*FRS1*	Beta subunit of cytoplasmic phenylalanyl-tRNA synthetase, forms a tetramer with Frs2p to generate active enzyme; sequence is evolutionarily distant from mitochondrial phenylalanyl-tRNA synthetase (Msf1p), but substrate binding is similar|Frs1p; protein coding	-2.54	-2.39
YEL054C	*RPL12A*	Protein component of the large (60S) ribosomal subunit, nearly identical to Rpl12Bp; rpl12a rpl12b double mutant exhibits slow growth and slow translation; has similarity to E. coli L11 and rat L12 ribosomal proteins|Rpl12ap; protein coding	-2.45	-3.09
YDL082W	*RPL13A*	Protein component of the large (60S) ribosomal subunit, nearly identical to Rpl13Bp; not essential for viability; has similarity to rat L13 ribosomal protein|Rpl13ap; protein coding	-3.24	-2.58
YPR043W	*RPL43A*	Protein component of the large (60S) ribosomal subunit, identical to Rpl43Bp and has similarity to rat L37a ribosomal protein; null mutation confers a dominant lethal phenotype|Rpl43ap; protein coding	-2.79	-2.69
YOL139C	*CDC33*	Cdc33p|Cytoplasmic mRNA cap binding protein; the eIF4E-cap complex is responsible for mediating cap-dependent mRNA translation via interactions with the translation initiation factor eIF4G (Tif4631p or Tif4632p); protein coding	-2.11	-1.68

YDL061C	*RPS29B*	Protein component of the small (40S) ribosomal subunit; nearly identical to Rps29Ap and has similarity to rat S29 and E. coli S14 ribosomal proteins|Rps29bp; protein coding	-2.48	-2.13
YOL127W	*RPL25*	Primary rRNA-binding ribosomal protein component of the large (60S) ribosomal subunit, has similarity to E. coli L23 and rat L23a ribosomal proteins; binds to 26S rRNA via a conserved C-terminal motif|Rpl25p; protein coding	-2.96	-2.38
YOL120C	*RPL18A*	Protein component of the large (60S) ribosomal subunit, identical to Rpl18Bp and has similarity to rat L18 ribosomal protein; intron of RPL18A pre-mRNA forms stem-loop structures that are a target for Rnt1p cleavage leading to degradation|Rpl18ap; protein coding	-3.67	-2.11
YOL097C	*WRS1*	Cytoplasmic tryptophanyl-tRNA synthetase, aminoacylates tryptophanyl-tRNA|Wrs1p; protein coding	-2.56	-2.19
**YORF**	***Gene***	**Description**	**Fold change *myo1*Δ**	**Fold change *chs2*Δ**
**Metabolism**				
YOL056W	*GPM3*	Gpm3p|Homolog of Gpm1p phosphoglycerate mutase, which converts 3-phosphoglycerate to 2-phosphoglycerate in glycolysis; may be non-functional derivative of a gene duplication event; protein coding	-1.48	-1.41
YGL257C	*MNT2*	Mannosyltransferase involved in adding the 4th and 5th mannose residues of O-linked glycans|Mnt2p; protein coding	-2.35	-1.80
YKR066C	*CCP1*	Ccp1p|Mitochondrial cytochrome-c peroxidase; degrades reactive oxygen species in mitochondria, involved in the response to oxidative stress; protein coding	6.02	-1.67
YML126C	*ERG13*	3-hydroxy-3-methylglutaryl-CoA (HMG-CoA) synthase, catalyzes the	-2.15	-1.41

		formation of HMG-CoA from acetyl-CoA and acetoacetyl-CoA; involved in the second step in mevalonate biosynthesis|Erg13p; protein coding		
YML120C	*NDI1*	NADH:ubiquinone oxidoreductase, transfers electrons from NADH to ubiquinone in the respiratory chain but does not pump protons, in contrast to the higher eukaryotic multisubunit respiratory complex I; phosphorylated; homolog of human AMID|Ndi1p; protein coding	2.15	2.45
YML106W	*URA5*	One of two orotate phosphoribosyltransferase isozymes (see also URA10) that catalyze the fifth enzymatic step in de novo biosynthesis of pyrimidines, converting orotate into orotidine-5'-phosphate|Ura5p; protein coding	-3.45	-2.48
YDR380W	*ARO10*	Aro10p|Phenylpyruvate decarboxylase, catalyzes decarboxylation of phenylpyruvate to phenylacetaldehyde, which is the first specific step in the Ehrlich pathway; protein coding	-3.58	3.54
YKL211C	*TRP3*	Bifunctional enzyme exhibiting both indole-3-glycerol-phosphate synthase and anthranilate synthase activities, forms multifunctional hetero-oligomeric anthranilate synthase:indole-3-glycerol phosphate synthase enzyme complex with Trp2p|Trp3p; protein coding	-1.70	2.12
YNR019W	*ARE2*	Acyl-CoA:sterol acyltransferase, isozyme of Are1p; endoplasmic reticulum enzyme that contributes the major sterol esterification activity in the presence of oxygen|Are2p; protein coding	1.41	2.23
YGL148W	*ARO2*	Aro2p|Bifunctional chorismate synthase and flavin reductase, catalyzes the conversion of 5-enolpyruvylshikimate 3-phosphate (EPSP) to form chorismate, which is a precursor to aromatic amino acids; protein coding	-2.57	-2.09

YIL045W	*PIG2*	Pig2p|Putative type-1 protein phosphatase targeting subunit that tethers Glc7p type-1 protein phosphatase to Gsy2p glycogen synthase; protein coding	6.76	9.00
YDR321W	*ASP1*	Asp1p|Cytosolic L-asparaginase, involved in asparagine catabolism; protein coding	-2.06	-2.95
YKL150W	*MCR1*	Mcr1p|Mitochondrial NADH-cytochrome b5 reductase, involved in ergosterol biosynthesis; protein coding	6.11	2.29
YML008C	*ERG6*	Delta(24)-sterol C-methyltransferase, converts zymosterol to fecosterol in the ergosterol biosynthetic pathway by methylating position C-24; localized to both lipid particles and mitochondrial outer membrane|Erg6p; protein coding	-2.20	-1.29
YHR193C	*EGD2*	Alpha subunit of the heteromeric nascent polypeptide-associated complex (NAC) involved in protein sorting and translocation, associated with cytoplasmic ribosomes|Egd2p; protein coding	-1.87	-1.80
YBR252W	*DUT1*	Dut1p|dUTPase, catalyzes hydrolysis of dUTP to dUMP and PPi, thereby preventing incorporation of uracil into DNA during replication; critical for the maintenance of genetic stability; protein coding	-2.39	-1.78
YBR249C	*ARO4*	3-deoxy-D-arabino-heptulosonate-7-phosphate (DAHP) synthase, catalyzes the first step in aromatic amino acid biosynthesis and is feedback-inhibited by tyrosine or high concentrations of phenylalanine or tryptophan|Aro4p; protein coding	-2.67	-3.59
YBR248C	*HIS7*	His7p|Imidazole glycerol phosphate synthase (glutamine amidotransferase:cyclase), catalyzes the fifth and sixth steps of histidine biosynthesis and also produces 5-aminoimidazole-4-carboxamide ribotide (AICAR), a purine precursor; protein coding	-2.02	-1.45

YGL022W	*STT3*	Stt3p|Subunit of the oligosaccharyltransferase complex of the ER lumen, which catalyzes asparagine-linked glycosylation of newly synthesized proteins; forms a subcomplex with Ost3p and Ost4p and is directly involved in catalysis; protein coding	-1.75	-1.24
YDR231C	*COX20*	Cox20p|Mitochondrial inner membrane protein, required for proteolytic processing of Cox2p and its assembly into cytochrome c oxidase; protein coding	2.15	2.18
YGL012W	*ERG4*	C-24(28) sterol reductase, catalyzes the final step in ergosterol biosynthesis; mutants are viable, but lack ergosterol|Erg4p; protein coding	-2.54	-2.16
YOR377W	*ATF1*	Alcohol acetyltransferase with potential roles in lipid and sterol metabolism; responsible for the major part of volatile acetate ester production during fermentation|Atf1p; protein coding	2.20	2.32
YHR128W	*FUR1*	Fur1p|Uracil phosphoribosyltransferase, synthesizes UMP from uracil; involved in the pyrimidine salvage pathway; protein coding	-2.72	-2.75
YHR123W	*EPT1*	Ept1p|sn-1,2-diacylglycerol ethanolamine- and cholinephosphotranferase; not essential for viability; protein coding	-1.90	-1.25
YBR183W	*YPC1*	Alkaline ceramidase that also has reverse (CoA-independent) ceramide synthase activity, catalyzes both breakdown and synthesis of phytoceramide; overexpression confers fumonisin B1 resistance|Ypc1p; protein coding	2.89	1.48
YNL153C	*GIM3*	Gim3p|Subunit of the heterohexameric cochaperone prefoldin complex which binds specifically to cytosolic chaperonin and transfers target proteins to it; protein coding	-2.06	-1.30

YBR117C	*TKL2*	Tkl2p|Transketolase, similar to Tkl1p; catalyzes conversion of xylulose-5-phosphate and ribose-5-phosphate to sedoheptulose-7-phosphate and glyceraldehyde-3-phosphate in the pentose phosphate pathway; needed for synthesis of aromatic amino acids; protein coding	14.35	1.27
YJR143C	*PMT4*	Pmt4p|Protein O-mannosyltransferase, transfers mannose residues from dolichyl phosphate-D-mannose to protein serine/threonine residues; appears to form homodimers in vivo and does not complex with other Pmt proteins; target for new antifungals; protein coding	-2.83	-2.43
YJR121W	*ATP2*	Atp2p|Beta subunit of the F1 sector of mitochondrial F1F0 ATP synthase, which is a large, evolutionarily conserved enzyme complex required for ATP synthesis; phosphorylated; protein coding	2.76	2.88
YOR236W	*DFR1*	Dfr1p|Dihydrofolate reductase, part of the dTTP biosynthetic pathway, involved in folate metabolism, possibly required for mitochondrial function; protein coding	-2.77	-1.47
YLR251W	*SYM1*	Protein required for ethanol metabolism; induced by heat shock and localized to the inner mitochondrial membrane; homologous to mammalian peroxisomal membrane protein Mpv17|Sym1p; protein coding	3.30	1.91
YLR221C	*RSA3*	Protein with a likely role in ribosomal maturation, required for accumulation of wild-type levels of large (60S) ribosomal subunits; binds to the helicase Dbp6p in pre-60S ribosomal particles in the nucleolus|Rsa3p; protein coding	-1.90	-1.17
YGR285C	*ZUO1*	Cytosolic ribosome-associated chaperone that acts, together with Ssz1p and the Ssb proteins, as a chaperone for nascent polypeptide	-1.79	1.69

		chains; contains a DnaJ domain and functions as a J-protein partner for Ssb1p and Ssb2p|Zuo1p; protein coding		
YGR256W	*GND2*	6-phosphogluconate dehydrogenase (decarboxylating), catalyzes an NADPH regenerating reaction in the pentose phosphate pathway; required for growth on D-glucono-delta-lactone|Gnd2p; protein coding	9.92	3.06
YGR248W	*SOL4*	6-phosphogluconolactonase with similarity to Sol3p|Sol4p; protein coding	15.32	2.97
YMR300C	*ADE4*	Ade4p|Phosphoribosylpyrophosphate amidotransferase (PRPPAT; amidophosphoribosyltransferase), catalyzes first step of the 'de novo' purine nucleotide biosynthetic pathway; protein coding	-1.78	2.17
YMR274C	*RCE1*	Rce1p|Type II CAAX prenyl protease involved in the proteolysis and maturation of Ras and the a-factor mating pheromone; protein coding	-1.81	-1.26
YDL205C	*HEM3*	Hem3p|Phorphobilinogen deaminase, catalyzes the conversion of 4-porphobilinogen to hydroxymethylbilane, the third step in the heme biosynthetic pathway; localizes to both the cytoplasm and nucleus; expression is regulated by Hap2p-Hap3p; protein coding	1.96	3.15
YER055C	*HIS1*	ATP phosphoribosyltransferase, a hexameric enzyme, catalyzes the first step in histidine biosynthesis; mutations cause histidine auxotrophy and sensitivity to Cu, Co, and Ni salts; transcription is regulated by general amino acid control|His1p; protein coding	-2.32	-2.05
YER043C	*SAH1*	S-adenosyl-L-homocysteine hydrolase, catabolizes S-adenosyl-L-homocysteine which is formed after donation of the activated methyl group of S-adenosyl-L-methionine (AdoMet) to an acceptor|Sah1p; protein coding	-2.78	-1.26

YJL155C	*FBP26*	Fbp26p|Fructose-2,6-bisphosphatase, required for glucose metabolism; protein coding	2.09	1.93
YMR250W	*GAD1*	Gad1p|Glutamate decarboxylase, converts glutamate into gamma-aminobutyric acid (GABA) during glutamate catabolism; involved in response to oxidative stress; protein coding	7.09	10.78
YPR145W	*ASN1*	Asn1p|Asparagine synthetase, isozyme of Asn2p; catalyzes the synthesis of L-asparagine from L-aspartate in the asparagine biosynthetic pathway; protein coding	-4.10	-1.73
YBL015W	*ACH1*	Acetyl-coA hydrolase, primarily localized to mitochondria; phosphorylated; required for acetate utilization and for diploid pseudohyphal growth|Ach1p; protein coding	1.93	6.16
YER023W	*PRO3*	Delta 1-pyrroline-5-carboxylate reductase, catalyzes the last step in proline biosynthesis|Pro3p; protein coding	-2.07	1.36
YLR100W	*ERG27*	3-keto sterol reductase, catalyzes the last of three steps required to remove two C-4 methyl groups from an intermediate in ergosterol biosynthesis; mutants are sterol auxotrophs|Erg27p; protein coding	-2.59	-1.34
YDL141W	*BPL1*	Biotin:apoprotein ligase, covalently modifies proteins with the addition of biotin, required for acetyl-CoA carboxylase (Acc1p) holoenzyme formation|Bpl1p; protein coding	-2.08	-1.32
YAL060W	*BDH1*	Bdh1p|NAD-dependent (2R,3R)-2,3-butanediol dehydrogenase, a zinc-containing medium-chain alcohol dehydrogenase, produces 2,3-butanediol from acetoin during fermentation and allows using 2,3-butanediol as a carbon source during aerobic growth; protein coding	5.86	2.67
YDL103C	*QRI1*	Qri1p|UDP-N-acetylglucosamine pyrophosphorylase, catalyzes the formation of UDP-N-	-2.45	-1.56

		acetylglucosamine (UDP-GlcNAc), which is important in cell wall biosynthesis, protein N-glycosylation, and GPI anchor biosynthesis; protein coding		
YMR165C	*SMP2*	Mg2+-dependent phosphatidate (PA) phosphatase, catalyzes the dephosphorylation of PA to yield diacylglycerol and Pi, responsible for de novo lipid synthesis; homologous to mammalian lipin 1|Pah1p; protein coding	2.188	1.86
YLR017W	*MEU1*	Methylthioadenosine phosphorylase (MTAP), catalyzes the initial step in the methionine salvage pathway; affects polyamine biosynthesis through regulation of ornithine decarboxylase (Spe1p) activity; regulates ADH2 gene expression|Meu1p; protein coding	-1.64	-1.64
YGR078C	*PAC10*	Pac10p|Part of the heteromeric co-chaperone GimC/prefoldin complex, which promotes efficient protein folding; protein coding	-2.01	1.25
YDR518W	*EUG1*	Eug1p|Protein disulfide isomerase of the endoplasmic reticulum lumen, function overlaps with that of Pdi1p; may interact with nascent polypeptides in the ER; protein coding	-1.82	-1.53
**YORF**	**Gene**	**Description**	**Fold change *myo1*Δ**	**Fold change *chs2*Δ**
**Stress response**				
YCR104W	*PAU3*	Part of 23-member seripauperin multigene family encoded mainly in subtelomeric regions, active during alcoholic fermentation, regulated by anaerobiosis, negatively regulated by oxygen, repressed by heme|Pau3p; protein coding	1.75	2.25
YGR008C	*STF2*	Protein involved in regulation of the mitochondrial F1F0-ATP synthase; Stf1p and Stf2p act as stabilizing factors that enhance inhibitory action of the Inh1p protein|Stf2p; protein	7.29	3.24

		coding		
YOL052C-A	*DDR2*	Ddr2p|Multistress response protein, expression is activated by a variety of xenobiotic agents and environmental or physiological stresses; protein coding	3.30	2.12
YPL223C	*GRE1*	Gre1p|Hydrophilin of unknown function; stress induced (osmotic, ionic, oxidative, heat shock and heavy metals); regulated by the HOG pathway; protein coding	9.48	1.68
YKR072C	*SIS2*	Negative regulatory subunit of the protein phosphatase 1 Ppz1p; involved in ion homeostasis and cell cycle progression|Sis2p; protein coding	1.89	-1.15
YIL101C	*XBP1*	Transcriptional repressor that binds to promoter sequences of the cyclin genes, CYS3, and SMF2; expression is induced by stress or starvation during mitosis, and late in meiosis; member of the Swi4p/Mbp1p family; potential Cdc28p substrate|Xbp1p; protein coding	2.29	1.95
YIL053W	*RHR2*	Constitutively expressed isoform of DL-glycerol-3-phosphatase; involved in glycerol biosynthesis, induced in response to both anaerobic and, along with the Hor2p/Gpp2p isoform, osmotic stress|Rhr2p; protein coding	-2.58	-1.59
YKL201C	*MNN4*	Mnn4p|Putative positive regulator of mannosylphosphate transferase (Mnn6p), involved in mannosylphosphorylation of N-linked oligosaccharides; expression increases in late-logarithmic and stationary growth phases; protein coding	2.53	3.12
YML070W	*DAK1*	Dak1p|Dihydroxyacetone kinase, required for detoxification of dihydroxyacetone (DHA); involved in stress adaptation; protein coding	5.87	1.37
YDR214W	*AHA1*	Aha1p|Co-chaperone that binds to Hsp82p and activates its ATPase activity; similar to Hch1p; expression is regulated by stresses such as heat	3.85	1.39

		shock; protein coding		
YBR169C	*SSE2*	Member of the heat shock protein 70 (HSP70) family; may be involved in protein folding; localized to the cytoplasm; highly homologous to the heat shock protein Sse1p|Sse2p; protein coding	7.11	1.89
YHR104W	*GRE3*	Aldose reductase involved in methylglyoxal, d-xylose and arabinose metabolism; stress induced (osmotic, ionic, oxidative, heat shock, starvation and heavy metals); regulated by the HOG pathway|Gre3p; protein coding	4.11	2.06
YNL160W	*YGP1*	Cell wall-related secretory glycoprotein; induced by nutrient deprivation-associated growth arrest and upon entry into stationary phase; may be involved in adaptation prior to stationary phase entry; has similarity to Sps100p|Ygp1p; protein coding	7.55	2.15
YFL020C	*PAU5*	Part of 23-member seripauperin multigene family encoded mainly in subtelomeric regions, active during alcoholic fermentation, regulated by anaerobiosis, negatively regulated by oxygen, repressed by heme|Pau5p; protein coding	1.82	2.62
YFL014W	*HSP12*	Hsp12p|Plasma membrane localized protein that protects membranes from desiccation; induced by heat shock, oxidative stress, osmostress, stationary phase entry, glucose depletion, oleate and alcohol; regulated by the HOG and Ras-Pka pathways; protein coding	36.77	17.86
YFL010C	*WWM1*	WW domain containing protein of unknown function; binds to Mca1p, a caspase-related protease that regulates H2O2-induced apoptosis; overexpression causes Gi phase growth arrest and clonal death that is suppressed by overexpression of MCA1|Wwm1p; protein coding	-1.71	1.16

YBR072W	*HSP26*	Hsp26p|Small heat shock protein (sHSP) with chaperone activity; forms hollow, sphere-shaped oligomers that suppress unfolded proteins aggregation; oligomer activation requires a heat-induced conformational change; not expressed in unstressed cells; protein coding	23.32	19.36
YMR251W-A	*HOR7*	Hor7p|Protein of unknown function; overexpression suppresses Ca2+ sensitivity of mutants lacking inositol phosphorylceramide mannosyltransferases Csg1p and Csh1p; transcription is induced under hyperosmotic stress and repressed by alpha factor; protein coding	9.17	14.53
YAR020C	*PAU7*	Part of 23-member seripauperin multigene family, active during alcoholic fermentation, regulated by anaerobiosis, inhibited by oxygen, repressed by heme|Pau7p; protein coding	1.79	2.55
YMR169C	*ALD3*	Ald3p|Cytoplasmic aldehyde dehydrogenase, involved in beta-alanine synthesis; uses NAD+ as the preferred coenzyme; very similar to Ald2p; expression is induced by stress and repressed by glucose; protein coding	41.51	2.68
YGR088W	*CTT1*	Ctt1p|Cytosolic catalase T, has a role in protection from oxidative damage by hydrogen peroxide; protein coding	26.81	1.63
**YORF**	***Gene***	**Description**	**Fold change *myo1*Δ**	**Fold change *chs2*Δ**
**Transport**				
YPL234C	*TFP3*	Tfp3p|Vacuolar ATPase V0 domain subunit c', involved in proton transport activity; hydrophobic integral membrane protein (proteolipid) containing four transmembrane segments; N and C termini are in the vacuolar lumen; protein coding	-1.41	1.70
YPL134C	*ODC1*	Mitochondrial inner membrane transporter, exports 2-oxoadipate and	1.96	1.52

		2-oxoglutarate from the mitochondrial matrix to the cytosol for lysine and glutamate biosynthesis and lysine catabolism; suppresses, in multicopy, an fmc1 null mutation|Odc1p; protein coding		
YKL188C	*PXA2*	Pxa2p|Subunit of a heterodimeric peroxisomal ATP-binding cassette transporter complex (Pxa1p-Pxa2p), required for import of long-chain fatty acids into peroxisomes; similarity to human adrenoleukodystrophy transporter and ALD-related proteins; protein coding	2.45	2.08
YML054C	*CYB2*	Cyb2p|Cytochrome b2 (L-lactate cytochrome-c oxidoreductase), component of the mitochondrial intermembrane space, required for lactate utilization; expression is repressed by glucose and anaerobic conditions; protein coding	5.44	2.47
YDR276C	*PMP3*	Pmp3p|Small plasma membrane protein related to a family of plant polypeptides that are overexpressed under high salt concentration or low temperature, not essential for viability, deletion causes hyperpolarization of the plasma membrane potential; protein coding	2.62	1.52
YGL006W	*PMC1*	Pmc1p|Vacuolar Ca2+ ATPase involved in depleting cytosol of Ca2+ ions; prevents growth inhibition by activation of calcineurin in the presence of elevated concentrations of calcium; similar to mammalian PMCA1a; protein coding	2.29	1.54
YFR053C	*HXK1*	Hexokinase isoenzyme 1, a cytosolic protein that catalyzes phosphorylation of glucose during glucose metabolism; expression is highest during growth on non-glucose carbon sources; glucose-induced repression involves the hexokinase Hxk2p|Hxk1p; protein coding	14.16	9.38
YFR033C	*QCR6*	Qcr6p|Subunit 6 of the ubiquinol cytochrome-c reductase complex,	2.34	2.29

		which is a component of the mitochondrial inner membrane electron transport chain; highly acidic protein; required for maturation of cytochrome c1; protein coding		
YOR317W	*FAA1*	Faa1p|Long chain fatty acyl-CoA synthetase with a preference for C12:0-C16:0 fatty acids; involved in the activation of imported fatty acids; localized to both lipid particles and mitochondrial outer membrane; essential for stationary phase; protein coding	2.81	2.43
YNL142W	*MEP2*	Ammonium permease involved in regulation of pseudohyphal growth; belongs to a ubiquitous family of cytoplasmic membrane proteins that transport only ammonium (NH4+); expression is under the nitrogen catabolite repression regulation|Mep2p; protein coding	-3.16	-1.72
YBR043C	*QDR3*	Multidrug transporter of the major facilitator superfamily, required for resistance to quinidine, barban, cisplatin, and bleomycin|Qdr3p; protein coding	2.69	1.22
YOR216C	*RUD3*	Golgi matrix protein involved in the structural organization of the cis-Golgi; interacts genetically with COG3 and USO1|Rud3p; protein coding	-2.43	-1.54
YDL234C	*GYP7*	GTPase-activating protein for yeast Rab family members including: Ypt7p (most effective), Ypt1p, Ypt31p, and Ypt32p (in vitro); involved in vesicle mediated protein trafficking|Gyp7p; protein coding	1.93	1.74
YBL045C	*COR1*	Cor1p|Core subunit of the ubiquinol-cytochrome c reductase complex (bc1 complex), which is a component of the mitochondrial inner membrane electron transport chain; protein coding	2.67	2.60
YJL166W	*QCR8*	Qcr8p|Subunit 8 of ubiquinol cytochrome-c reductase complex, which is a component of the	2.69	1.23

		mitochondrial inner membrane electron transport chain; oriented facing the intermembrane space; expression is regulated by Abf1p and Cpf1p; protein coding		
YGR172C	*YIP1*	Integral membrane protein required for the biogenesis of ER-derived COPII transport vesicles; interacts with Yif1p and Yos1p; localizes to the Golgi, the ER, and COPII vesicles|Yip1p; protein coding	-1.63	-1.69
YEL039C	*CYC7*	Cyc7p|Cytochrome c isoform 2, expressed under hypoxic conditions; electron carrier of the mitochondrial intermembrane space that transfers electrons from ubiquinone-cytochrome c oxidoreductase to cytochrome c oxidase during cellular respiration; protein coding	5.52	1.17
YEL031W	*SPF1*	P-type ATPase, ion transporter of the ER membrane involved in ER function and Ca2+ homeostasis; required for regulating Hmg2p degradation; confers sensitivity to a killer toxin (SMKT) produced by Pichia farinosa KK1|Spf1p; protein coding	-2.33	-1.73
YAL022C	*FUN26*	Fun26p|Nucleoside transporter with broad nucleoside selectivity; localized to intracellular membranes; protein coding	-1.67	-1.14
YDR534C	*FIT1*	Fit1p|Mannoprotein that is incorporated into the cell wall via a glycosylphosphatidylinositol (GPI) anchor, involved in the retention of siderophore-iron in the cell wall; protein coding	-4.60	1.17
YLL043W	*FPS1*	Fps1p|Plasma membrane glycerol channel, member of the major intrinsic protein (MIP) family of channel proteins; involved in efflux of glycerol and in uptake of the trivalent metalloids arsenite and antimonite; protein coding	2.79	1.94
YDR513W	*TTR1*	Cytoplasmic glutaredoxin, thioltransferase, glutathione-	1.74	1.39

		dependent disulfide oxidoreductase involved in maintaining redox state of target proteins, also exhibits glutathione peroxidase activity, expression induced in response to stress|Grx2p; protein coding		
**YORF**	**Gene**	**Description**	**Fold change *myo1*Δ**	**Fold change *chs2*Δ**
**Carbohydrate metabolism**				
YIL155C	*GUT2*	Gut2p|Mitochondrial glycerol-3-phosphate dehydrogenase; expression is repressed by both glucose and cAMP and derepressed by non-fermentable carbon sources in a Snf1p, Rsf1p, Hap2/3/4/5 complex dependent manner; protein coding	3.77	11.97
YKR058W	*GLG1*	Glg1p|Self-glucosylating initiator of glycogen synthesis, also glucosylates n-dodecyl-beta-D-maltoside; similar to mammalian glycogenin; protein coding	4.55	1.93
YNR034W	*SOL1*	Protein with a possible role in tRNA export; shows similarity to 6-phosphogluconolactonase non-catalytic domains but does not exhibit this enzymatic activity; homologous to Sol2p, Sol3p, and Sol4p|Sol1p; protein coding	2.09	1.22
YGL156W	*AMS1*	Ams1p|Vacuolar alpha mannosidase, involved in free oligosaccharide (fOS) degradation; delivered to the vacuole in a novel pathway separate from the secretory pathway; protein coding	2.77	8.38
YCL040W	*GLK1*	Glk1p|Glucokinase, catalyzes the phosphorylation of glucose at C6 in the first irreversible step of glucose metabolism; one of three glucose phosphorylating enzymes; expression regulated by non-fermentable carbon sources; protein coding	6.82	5.55
YKL127W	*PGM1*	Pgm1p|Phosphoglucomutase, minor isoform; catalyzes the conversion from glucose-1-phosphate to glucose-	-2.68	-1.92

		6-phosphate, which is a key step in hexose metabolism; protein coding		
YGL028C	*SCW11*	Cell wall protein with similarity to glucanases; may play a role in conjugation during mating based on its regulation by Ste12p|Scw11p; protein coding	-1.89	1.09
YKL035W	*UGP1*	UDP-glucose pyrophosphorylase (UGPase), catalyses the reversible formation of UDP-Glc from glucose 1-phosphate and UTP, involved in a wide variety of metabolic pathways, expression modulated by Pho85p through Pho4p|Ugp1p; protein coding	9.13	7.22
YBR126C	*TPS1*	Synthase subunit of trehalose-6-phosphate synthase/phosphatase complex, which synthesizes the storage carbohydrate trehalose; also found in a monomeric form; expression is induced by the stress response and repressed by the Ras-cAMP pathway|Tps1p; protein coding	5.30	3.78
YDR074W	*TPS2*	Phosphatase subunit of the trehalose-6-phosphate synthase/phosphatase complex, which synthesizes the storage carbohydrate trehalose; expression is induced by stress conditions and repressed by the Ras-cAMP pathway|Tps2p; protein coding	7.47	2.84
YGR282C	*BGL2*	Bgl2p|Endo-beta-1,3-glucanase, major protein of the cell wall, involved in cell wall maintenance; protein coding	2.08	2.74
YDR001C	*NTH1*	Neutral trehalase, degrades trehalose; required for thermotolerance and may mediate resistance to other cellular stresses; may be phosphorylated by Cdc28p|Nth1p; protein coding	5.35	1.69
YMR305C	*SCW10*	Cell wall protein with similarity to glucanases; may play a role in conjugation during mating based on mutant phenotype and its regulation by Ste12p|Scw10p; protein coding	-4.20	-2.27

YPR184W	*GDB1*	Gdb1p|Glycogen debranching enzyme containing glucanotranferase and alpha-1,6-amyloglucosidase activities, required for glycogen degradation; phosphorylated in mitochondria; protein coding	7.27	3.56
YER054C	*GIP2*	Gip2p|Putative regulatory subunit of the protein phosphatase Glc7p, involved in glycogen metabolism; contains a conserved motif (GVNK motif) that is also found in Gac1p, Pig1p, and Pig2p; protein coding	3.90	2.11
YPR160W	*GPH1*	Gph1p|Non-essential glycogen phosphorylase required for the mobilization of glycogen, activity is regulated by cyclic AMP-mediated phosphorylation, expression is regulated by stress-response elements and by the HOG MAP kinase pathway; protein coding	18.84	29.41
YER003C	*PMI40*	Mannose-6-phosphate isomerase, catalyzes the interconversion of fructose-6-P and mannose-6-P; required for early steps in protein mannosylation|Pmi40p; protein coding	-2.03	-2.31
YGR143W	*SKN1*	Protein involved in sphingolipid biosynthesis; type II membrane protein with similarity to Kre6p|Skn1p; protein coding	2.31	1.08
YEL040W	*UTR2*	Cell wall protein that functions in the transfer of chitin to beta(1-6)glucan; putative chitin transglycosidase; glycosylphosphatidylinositol (GPI)-anchored protein localized to the bud neck; has a role in cell wall maintenance|Utr2p; protein coding	-4.13	1.04
YEL011W	*GLC3*	Glc3p|Glycogen branching enzyme, involved in glycogen accumulation; green fluorescent protein (GFP)-fusion protein localizes to the cytoplasm in a punctate pattern; protein coding	7.34	36.62
YAL017W	*PSK1*	One of two (see also PSK2) PAS domain containing S/T protein kinases; coordinately regulates	1.61	1.66

		protein synthesis and carbohydrate metabolism and storage in response to a unknown metabolite that reflects nutritional status|Psk1p; protein coding		
**YORF**	***Gene***	**Description**	**Fold change *myo1*Δ**	**Fold change *chs2*Δ**
**Protein degradation**				
YLR120C	*YPS1*	Aspartic protease, attached to the plasma membrane via a glycosylphosphatidylinositol (GPI) anchor|Yps1p; protein coding	1.62	1.29
YMR174C	*PAI3*	Cytoplasmic proteinase A (Pep4p) inhibitor, dependent on Pbs2p and Hog1p protein kinases for osmotic induction; intrinsically unstructured, N-terminal half becomes ordered in the active site of proteinase A upon contact|Pai3p; protein coding	4.28	1.64
YEL060C	*PRB1*	Prb1p|Vacuolar proteinase B (yscB), a serine protease of the subtilisin family; involved in protein degradation in the vacuole and required for full protein degradation during sporulation; protein coding	2.62	4.89
YPR191W	*QCR2*	Qcr2p|Subunit 2 of the ubiquinol cytochrome-c reductase complex, which is a component of the mitochondrial inner membrane electron transport chain; phosphorylated; transcription is regulated by Hap1p, Hap2p/Hap3p, and heme; protein coding	3.14	3.81
YMR304W	*UBP15*	Ubiquitin-specific protease that may play a role in ubiquitin precursor processing|Ubp15p; protein coding	1.47	1.06
YOR219C	*STE13*	Dipeptidyl aminopeptidase, Golgi integral membrane protein that cleaves on the carboxyl side of repeating -X-Ala- sequences, required for maturation of alpha factor, transcription is induced by a-factor|Ste13p; protein coding	1.68	1.07

YBR105C	*VID24*	Peripheral membrane protein located at Vid (vacuole import and degradation) vesicles; regulates fructose-1,6-bisphosphatase (FBPase) targeting to the vacuole; involved in proteasome-dependent catabolite degradation of FBPase|Vid24p; protein coding	2.41	-1.18
YKL034W	*TUL1*	Golgi-localized RING-finger ubiquitin ligase (E3), involved in ubiquitinating and sorting membrane proteins that contain polar transmembrane domains to multivesicular bodies for delivery to the vacuole for quality control purposes|Tul1p; protein coding	1.93	1.10
YGL227W	*VID30*	Protein involved in proteasome-dependent catabolite degradation of fructose-1,6-bisphosphatase (FBPase); shifts the balance of nitrogen metabolism toward the production of glutamate; localized to the nucleus and the cytoplasm|Vid30p; protein coding	1.73	1.34
**YORF**	***Gene***	**Description**	**Fold change *myo1*Δ**	**Fold change *chs2*Δ**
**Transcription**				
YDL150W	*RPC53*	RNA polymerase III subunit C53|Rpc53p; protein coding	-1.74	-1.95
YPR187W	*RPO26*	RNA polymerase subunit ABC23, common to RNA polymerases I, II, and III; part of central core; similar to bacterial omega subunit|Rpo26p; protein coding	-2.18	-2.13
YLR228C	*ECM22*	Ecm22p|Sterol regulatory element binding protein, regulates transcription of the sterol biosynthetic genes ERG2 and ERG3; member of the fungus-specific Zn[2]-Cys[6] binuclear cluster family of transcription factors; homologous to Upc2p; protein coding	1.68	4.53
YKL185W	*ASH1*	Ash1p|Zinc-finger inhibitor of HO transcription; mRNA is localized and translated in the distal tip of anaphase	-2.05	1.73

		cells, resulting in accumulation of Ash1p in daughter cell nuclei and inhibition of HO expression; potential Cdc28p substrate; protein coding		
YOL005C	*RPB11*	RNA polymerase II subunit B12.5; part of central core; similar to Rpc19p and bacterial alpha subunit|Rpb11p; protein coding	-2.02	-1.17
**YORF**	**Gene**	**Description**	**Fold change *myo1*Δ**	**Fold change *chs2*Δ**
**DNA metabolism**				
YJL080C	*SCP160*	Essential RNA-binding G protein effector of mating response pathway, mainly associated with nuclear envelope and ER, interacts in mRNA-dependent manner with translating ribosomes via multiple KH domains, similar to vertebrate vigilins|Scp160p; protein coding	-2.64	-2.29
YDL227C	*HO*	Hop|Site-specific endonuclease required for gene conversion at the MAT locus (homothallic switching) through the generation of a ds DNA break; expression restricted to mother cells in late G1 as controlled by Swi4p-Swi6p, Swi5p and Ash1p; protein coding	-4.15	-1.80
YER120W	*SCS2*	Integral ER membrane protein that regulates phospholipid metabolism via an interaction with the FFAT motif of Opi1p, also involved in telomeric silencing, disruption causes inositol auxotrophy above 34 degrees C, VAP homolog|Scs2p; protein coding	-1.54	-1.44
YOR191W	*RIS1*	Member of the SWI/SNF family of DNA-dependent ATPases, plays a role in antagonizing silencing during mating-type switching, contains an N-terminal domain that interacts with Sir4p and a C-terminal SNF2 domain|Ris1p; protein coding	1.49	1.13

YNL030W	*HHF2*	Hhf2p|One of two identical histone H4 proteins (see also HHF1); core histone required for chromatin assembly and chromosome function; contributes to telomeric silencing; N-terminal domain involved in maintaining genomic integrity; protein coding	-1.89	-1.24
YER171W	*RAD3*	5' to 3' DNA helicase, involved in nucleotide excision repair and transcription; subunit of RNA polymerase II transcription initiation factor TFIIH; subunit of Nucleotide Excision Repair Factor 3 (NEF3); homolog of human XPD protein|Rad3p; protein coding	-1.75	-1.52
YDR097C	*MSH6*	Msh6p|Protein required for mismatch repair in mitosis and meiosis, forms a complex with Msh2p to repair both single-base & insertion-deletion mispairs; potentially phosphorylated by Cdc28p; protein coding	-2.21	-2.03
YBR214W	*SDS24*	One of two S. cerevisiae homologs (Sds23p and Sds24p) of the Schizosaccharomyces pombe Sds23 protein, which genetic studies have implicated in APC/cyclosome regulation; may play an indirect role in fluid-phase endocytosis|Sds24p; protein coding	2.59	8.02
YBR245C	*ISW1*	Isw1p|Member of the imitation-switch (ISWI) class of ATP-dependent chromatin remodeling complexes; ATPase that forms a complex with Ioc2p and Ioc4p to regulate transcription elongation, and a complex with Ioc3p to repress transcription initiation; protein coding	-1.65	-1.58
YML069W	*POB3*	Pob3p|Subunit of the heterodimeric FACT complex (Spt16p-Pob3p), which facilitates RNA Pol II transcription elongation through nucleosomes by destabilizing and then reassembling nucleosome structure; interacts with DNA	-1.79	-2.04

		polymerase alpha (Pol1p); protein coding		
YGL213C	*SKI8*	Protein involved in exosome mediated 3' to 5' mRNA degradation and translation inhibition of non-poly(A) mRNAs as well as double-strand break formation during meiotic recombination; required for repressing propagation of dsRNA viruses|Ski8p; protein coding	-1.49	-1.14
**YORF**	***Gene***	**Description**	**Fold change *myo1*Δ**	**Fold change *chs2*Δ**
**Cell cycle**				
YDL055C	*PSA1*	GDP-mannose pyrophosphorylase (mannose-1-phosphate guanyltransferase), synthesizes GDP-mannose from GTP and mannose-1-phosphate in cell wall biosynthesis; required for normal cell wall structure|Psa1p; protein coding	-3.51	3.43
YGR041W	*BUD9*	Bud9p|Protein involved in bud-site selection; diploid mutants display a unipolar budding pattern instead of the wild-type bipolar pattern, and bud at the distal pole; protein coding	-3.01	1.21
YMR135C	*GID8*	Gid8p|Protein of unknown function, involved in proteasome-dependent catabolite inactivation of fructose-1,6-bisphosphatase; contains LisH and CTLH domains, like Vid30p; dosage-dependent regulator of START; protein coding	-2.39	2.17
YAL034W-A	*MTW1*	Essential component of the MIND kinetochore complex (Mtw1p Including Nnf1p-Nsl1p-Dsn1p) which joins kinetochore subunits contacting DNA to those contacting microtubules; critical to kinetochore assembly|Mtw1p; protein coding	-1.68	-1.24
YLR353W	*BUD8*	Bud8p|Protein involved in bud-site selection; diploid mutants display a unipolar budding pattern instead of the wild-type bipolar pattern, and bud at the proximal pole; protein coding	-1.34	-1.20

YNL289W	*PCL1*	Pcl1p|Pho85 cyclin of the Pcl1,2-like subfamily, involved in entry into the mitotic cell cycle and regulation of morphogenesis, localizes to sites of polarized cell growth; protein coding	-3.21	2.08
YNR027W	*BUD17*	Bud17p|Protein involved in bud-site selection; diploid mutants display a random budding pattern instead of the wild-type bipolar pattern; protein coding	-1.85	2.26
**YORF**	**Gene**	**Description**	**Fold change *myo1*Δ**	**Fold change *chs2*Δ**
**Protein transport**				
YDR358W	*GGA1*	Gga1p|Golgi-localized protein with homology to gamma-adaptin, interacts with and regulates Arf1p and Arf2p in a GTP-dependent manner in order to facilitate traffic through the late Golgi; protein coding	3.35	3.93
YDR202C	*RAV2*	Rav2p|Subunit of RAVE (Rav1p, Rav2p, Skp1p), a complex that associates with the V1 domain of the vacuolar membrane (H+)-ATPase (V-ATPase) and promotes assembly and reassembly of the holoenzyme; protein coding	2.00	-1.09
YDR200C	*VPS64*	Cytoplasmic protein required for cytoplasm to vacuole targeting of proteins; forms a complex with Far3p, Far7p, Far10p, and Far11p that is involved in pheromone-induced cell cycle arrest; also localized to the endoplasmic reticulum membrane|Vps64p; protein coding	1.64	1.92
YFL045C	*SEC53*	Phosphomannomutase, involved in synthesis of GDP-mannose and dolichol-phosphate-mannose; required for folding and glycosylation of secretory proteins in the ER lumen|Sec53p; protein coding	-2.67	-2.68
YJR135W-A	*TIM8*	Mitochondrial intermembrane space protein mediating import and insertion of polytopic inner	-2.50	-1.52

		membrane proteins; homolog of human DDP1 (deafness dystonia peptide 1) which is mutated in the X-linked Mohr-Tranebjaerg syndrome|Tim8p; protein coding		
YLR259C	*HSP60*	Hsp60p|Tetradecameric mitochondrial chaperonin required for ATP-dependent folding of precursor polypeptides and complex assembly; prevents aggregation and mediates protein refolding after heat shock; role in mtDNA transmission; phosphorylated; protein coding	2.83	-1.69
YBL075C	*SSA3*	ATPase involved in protein folding and the response to stress; plays a role in SRP-dependent cotranslational protein-membrane targeting and translocation; member of the heat shock protein 70 (HSP70) family; localized to the cytoplasm|Ssa3p; protein coding	3.812	1.23
YPR149W	*NCE102*	Nce102p|Protein of unknown function; contains transmembrane domains; involved in secretion of proteins that lack classical secretory signal sequences; component of the detergent-insoluble glycolipid-enriched complexes (DIGs); protein coding	6.22	8.35
YER008C	*SEC3*	Non-essential subunit of the exocyst complex (Sec3p, Sec5p, Sec6p, Sec8p, Sec10p, Sec15p, Exo70p, Exo84p) which mediates targeting of post-Golgi vesicles to sites of active exocytosis; Sec3p specifically is a spatial landmark for secretion|Sec3p; protein coding	1.49	-1.31
YLR066W	*SPC3*	Spc3p|Subunit of signal peptidase complex (Spc1p, Spc2p, Spc3p, Sec11p), which catalyzes cleavage of N-terminal signal sequences of proteins targeted to the secretory pathway; homologous to mammalian SPC22/23; protein coding	-2.20	-1.42
YGR082W	*TOM20*	Component of the TOM (translocase of outer membrane) complex	-1.82	-1.29

		responsible for recognition and initial import steps for all mitochondrially directed proteins; acts as a receptor for incoming precursor proteins|Tom20p; protein coding		
**YORF**	**Gene**	**Description**	**Fold change *myo1*Δ**	**Fold change *chs2*Δ**
**RNA processing**				
YDL051W	*LHP1*	Lhp1p|RNA binding protein required for maturation of tRNA and snRNA precursors; acts as a molecular chaperone for RNAs transcribed by polymerase III; homologous to human La (SS-B) autoantigen; protein coding	-2.05	-1.87
YGR129W	*SYF2*	Component of the spliceosome complex involved in pre-mRNA splicing; involved in regulation of cell cycle progression|Syf2p; protein coding	2.06	1.85
YLR107W	*REX3*	RNA exonuclease; required for maturation of the RNA component of RNase MRP; functions redundantly with Rnh70p and Rex2p in processing of U5 snRNA and RNase P RNA; member of RNase D family of exonucleases|Rex3p; protein coding	1.95	-1.29
YER032W	*FIR1*	Fir1p|Protein involved in 3' mRNA processing, interacts with Ref2p; potential Cdc28p substrate; protein coding	-1.74	2.52
YFL001W	*DEG1*	Deg1p|Non-essential tRNA:pseudouridine synthase, introduces pseudouridines at position 38 or 39 in tRNA, important for maintenance of translation efficiency and normal cell growth, localizes to both the nucleus and cytoplasm; protein coding	-1.36	1.42
YHR163W	*SOL3*	6-phosphogluconolactonase, catalyzes the second step of the pentose phosphate pathway; weak multicopy suppressor of los1-1 mutation; homologous to Sol2p and	-2.14	-1.27

		Sol1p|Sol3p; protein coding		
YIL038C	*NOT3*	Not3p|Subunit of the CCR4-NOT complex, which is a global transcriptional regulator with roles in transcription initiation and elongation and in mRNA degradation; protein coding	1.58	1.59
**YORF**	**Gene**	**Description**	**Fold change *myo1*Δ**	**Fold change *chs2*Δ**
**Signal transduction**				
YNL173C	*MDG1*	Mdg1p|Plasma membrane protein involved in G-protein mediated pheromone signaling pathway; overproduction suppresses bem1 mutations; protein coding	2.07	1.88
YDR085C	*AFR1*	Afr1p|Alpha-factor pheromone receptor regulator, negatively regulates pheromone receptor signaling; required for normal mating projection (shmoo) formation; required for Spa2p to recruit Mpk1p to shmoo tip during mating; interacts with Cdc12p; protein coding	3.65	1.44
YER118C	*SHO1*	Sho1p|Transmembrane osmosensor, participates in activation of both the Cdc42p- and MAP kinase-dependent filamentous growth pathway and the high-osmolarity glycerol response pathway; protein coding	-1.74	-1.02
YOR134W	*BAG7*	Bag7p|Rho GTPase activating protein (RhoGAP), stimulates the intrinsic GTPase activity of Rho1p, which plays a role in actin cytoskeleton organization and control of cell wall synthesis; structurally and functionally related to Sac7p; protein coding	1.89	2.59
YOR101W	*RAS1*	GTPase involved in G-protein signaling in the adenylate cyclase activating pathway, plays a role in cell proliferation; localized to the plasma membrane; homolog of mammalian RAS proto-oncogenes|Ras1p; protein coding	-2.29	-2.60

YJL005W	*CYR15*	Adenylate cyclase, required for cAMP production and cAMP-dependent protein kinase signaling; the cAMP pathway controls a variety of cellular processes, including metabolism, cell cycle, stress response, stationary phase, and sporulation|Cyr1p; protein coding	2.58	3.71
**YORF**	**Gene**	**Description**	**Fold change *myo1*Δ**	**Fold change *chs2*Δ**
**DNA replication**				
YDR054C	*CDC34*	Cdc34p|Ubiquitin-conjugating enzyme (E2) and catalytic subunit of SCF ubiquitin-protein ligase complex (together with Skp1p, Rbx1p, Cdc53p, and an F-box protein) that regulates cell cycle progression by targeting key substrates for degradation; protein coding	1.64	1.95
YOR074C	*CDC21*	Cdc21p|Thymidylate synthase, required for de novo biosynthesis of pyrimidine deoxyribonucleotides; expression is induced at G1/S; protein coding	-2.46	-1.40
**YORF**	**Gene**	**Description**	**Fold change *myo1*Δ**	**Fold change *chs2*Δ**
**Sporulation**				
YNL202W	*SPS19*	Peroxisomal 2,4-dienoyl-CoA reductase, auxiliary enzyme of fatty acid beta-oxidation; homodimeric enzyme required for growth and sporulation on petroselineate medium; expression induced during late sporulation and in the presence of oleate|Sps19p; protein coding		3.29 1.24
**YORF**	**Gene**	**Description**	**Fold change *myo1*Δ**	**Fold change *chs2*Δ**
DNA damage response				
YNL178W	*RPS3*	Protein component of the small (40S) ribosomal subunit, has apurinic/apyrimidinic (AP) endonuclease activity; essential for	-3.21	-2.81

		viability; has similarity to E. coli S3 and rat S3 ribosomal proteins|Rps3p; protein coding		
**Autophagy**				
YPL154C	*PEP4*	Pep4p|Vacuolar aspartyl protease (proteinase A), required for the posttranslational precursor maturation of vacuolar proteinases; important for protein turnover after oxidative damage; synthesized as a zymogen, self-activates; protein coding	3.16	5.40
YDL149W	*ATG9*	Transmembrane protein involved in forming Cvt and autophagic vesicles; cycles between the phagophore assembly site (PAS) and other cytosolic punctate structures, not found in autophagosomes; may be involved in membrane delivery to the PAS.	1.40	1.66
**DNA recombination**				
YML128C	*MSC1*	Msc1p|Protein of unknown function; mutant is defective in directing meiotic recombination events to homologous chromatids; the authentic, non-tagged protein is detected in highly purified mitochondria and is phosphorylated; protein coding	21.10	10.66
**Ribosome biogenesis**				
YOL040C	*RPS15*	Protein component of the small (40S) ribosomal subunit; has similarity to E. coli S19 and rat S15 ribosomal proteins|Rps15p; protein coding	-2.47	-2.64
**YORF**	**Gene**	**Description**	**Fold change *myo1*Δ**	**Fold change *chs2*Δ**
**DNA repair**				
YIR002C	*MPH1*	Member of the DEAH family of helicases, functions in an error-free DNA damage bypass pathway that involves homologous recombination, mutations confer a mutator phenotype|Mph1p; protein coding	1.97	1.71

YHR154W	*RTT107*	Protein implicated in Mms22-dependent DNA repair during S phase, DNA damage induces phosphorylation by Mec1p at one or more SQ/TQ motifs; interacts with Mms22p and Slx4p; has four BRCT domains; has a role in regulation of Ty1 transposition|Rtt107p; protein coding	-2.13	-1.21
YMR173W	*DDR48*	DNA damage-responsive protein, expression is increased in response to heat-shock stress or treatments that produce DNA lesions; contains multiple repeats of the amino acid sequence NNNDSYGS|Ddr48p; protein coding	4.10	2.67
**YORF**	**Gene**	**Description**	**Fold change *myo1*Δ**	**Fold change *chs2*Δ**
**Protein aminoacid phosphorylation**				
YLL019C	*KNS1*	Kns1p|Nonessential putative protein kinase of unknown cellular role; member of the LAMMER family of protein kinases, which are serine/threonine kinases also capable of phosphorylating tyrosine residues; protein coding	2.24	1.79
YJR059W	*PTK2*	Ptk2p|Putative serine/threonine protein kinase involved in regulation of ion transport across plasma membrane; enhances spermine uptake; protein coding	2.88	1.76
YJL164C	*TPK1*	Tpk1p|cAMP-dependent protein kinase catalytic subunit; promotes vegetative growth in response to nutrients via the Ras-cAMP signaling pathway; inhibited by regulatory subunit Bcy1p in the absence of cAMP; partially redundant with Tpk2p and Tpk3p; protein coding	2.35	2.25
YJL141C	*YAK1*	Serine-threonine protein kinase that is part of a glucose-sensing system involved in growth control in response to glucose availability;	2.46	1.21

		translocates from the cytoplasm to the nucleus and phosphorylates Pop2p in response to a glucose signal|Yak1p; protein coding		
YDR507C	*GIN4*	Gin4p|Protein kinase involved in bud growth and assembly of the septin ring, proposed to have kinase-dependent and kinase-independent activities; undergoes autophosphorylation; similar to Kcc4p and Hsl1p; protein coding	-1.94	-1.27
**YORF**	**Gene**	**Description**	**Fold change *myo1*Δ**	**Fold change *chs2*Δ**
**Unknown**				
YOL154W	*ZPS1*	Putative GPI-anchored protein; transcription is induced under low-zinc conditions, as mediated by the Zap1p transcription factor, and at alkaline pH|Zps1p; protein coding	-5.09	-3.74
ORF:YPR118W	user_defined	user_defined_node	-3.61	-2.24
ORF:YPR045C	user_defined	user_defined_node	2.52	1.52
ORF:YPL282C	user_defined	user_defined_node	2.00	2.12
ORF:YPL247C	user_defined	user_defined_node	5.51	1.42
ORF:YPL230W	user_defined	user_defined_node	10.66	3.01
ORF:YPL222W	user_defined	user_defined_node	4.34	2.33
ORF:YPL208W	user_defined	user_defined_node	-1.91	-1.62
ORF:YPL183C	user_defined	user_defined_node	-1.82	-2.20
ORF:YPL004C	user_defined	user_defined_node	5.26	1.56
ORF:YOR394W	user_defined	user_defined_node	1.88	2.28
ORF:YOR285W	user_defined	user_defined_node	2.73	2.25
ORF:YOR220W	user_defined	user_defined_node	3.05	4.70
ORF:YOR173W	user_defined	user_defined_node	10.14	3.38

ORF:YOR147W	user_defined	user_defined_node	1.89	2.37
ORF:YOR054C	user_defined	user_defined_node	2.88	2.22
ORF:YOR052C	user_defined	user_defined_node	1.49	1.35
YBR158W	*AMN1*	Amn1p|Protein required for daughter cell separation, multiple mitotic checkpoints, and chromosome stability; contains 12 degenerate leucine-rich repeat motifs; expression is induced by the Mitotic Exit Network (MEN); protein coding	41.50	2.68
YFR040W	*SAP155*	Protein that forms a complex with the Sit4p protein phosphatase and is required for its function; member of a family of similar proteins including Sap4p, Sap185p, and Sap190p|Sap155p; protein coding	2.46	-1.20
YBR302C	*COS2*	Cos2p|Protein of unknown function, member of the DUP380 subfamily of conserved, often subtelomerically-encoded proteins; protein coding	2.77	1.56
YNL336W	*COS1*	Cos1p|Protein of unknown function, member of the DUP380 subfamily of conserved, often subtelomerically-encoded proteins; protein coding	2.07	2.11
YML132W	*COS3*	Cos3p|Protein involved in salt resistance; interacts with sodium:hydrogen antiporter Nha1p; member of the DUP380 subfamily of conserved, often subtelomerically-encoded proteins; protein coding	2.97	1.59
YIL136W	*OM45*	Om45p|Protein of unknown function, major constituent of the mitochondrial outer membrane; located on the outer (cytosolic) face of the outer membrane; protein coding	9.36	4.09
YDR436W	*PPZ2*	Ppz2p|Serine/threonine protein phosphatase Z, isoform of Ppz1p; involved in regulation of potassium transport, which affects osmotic stability, cell cycle progression, and halotolerance; protein coding	1.69	1.06

YOL030W	*GAS5*	1,3-beta-glucanosyltransferase, has similarity to Gas1p; localizes to the cell wall|Gas5p; protein coding	1.32	-1.27
YDR451C	*YHP1*	One of two homeobox transcriptional repressors (see also Yox1p), that bind to Mcm1p and to early cell cycle box (ECB) elements of cell cycle regulated genes, thereby restricting ECB-mediated transcription to the M/G1 interval|Yhp1p; protein coding	-2.81	1.02
YOL084W	*PHM7*	Phm7p|Protein of unknown function, expression is regulated by phosphate levels; green fluorescent protein (GFP)-fusion protein localizes to the cell periphery and vacuole; protein coding	24.22	1.74
YHR076W	*PTC7*	Mitochondrially localized type 2C protein phosphatase; expression induced by growth on ethanol and by sustained osmotic stress; possible role in carbon source utilization in low oxygen environments|Ptc7p; protein coding	-1.83	-1.31
YFL062W	*COS4*	Cos4p|Protein of unknown function, member of the DUP380 subfamily of conserved, often subtelomerically-encoded proteins; protein coding	2.51	1.76
YJR127C	*ZMS1*	Rsf2p|Zinc-finger protein involved in transcriptional control of both nuclear and mitochondrial genes, many of which specify products required for glycerol-based growth, respiration, and other functions; protein coding	2.08	1.82
YOR276W	*CAF20*	Caf20p|Phosphoprotein of the mRNA cap-binding complex involved in translational control, repressor of cap-dependent translation initiation, competes with eIF4G for binding to eIF4E; protein coding	-1.95	-2.29
YNL074C	*MLF3*	Mlf3p|Serine-rich protein of unknown function; overproduction suppresses the growth inhibition caused by exposure to the immunosuppressant leflunomide; protein coding	1.60	3.17

YER150W	*SPI1*	GPI-anchored, serine/threonine rich cell wall protein of unknown function; basal expression requires Msn2p/Msn4p; expression is induced under conditions of stress and during the diauxic shift; similar to Sed1p|Spi1p; protein coding	10.46	1.79
YGR295C	*COS6*	Cos6p|Protein of unknown function, member of the DUP380 subfamily of conserved, often subtelomerically-encoded proteins; protein coding	2.01	2.76
YLR183C	*TOS4*	Tos4p|Transcription factor that binds to a number of promoter regions, particularly promoters of some genes involved in pheromone response and cell cycle; potential Cdc28p substrate; expression is induced in G1 by bound SBF; protein coding	-2.48	-1.26
BUD28	user_defined	user_defined_node	-2.05	-1.29
IMD1	user_defined	user_defined_node	-2.88	-3.23
ORF:YAL061W	user_defined	user_defined_node	8.92	10.34
ORF:YBL009W	user_defined	user_defined_node	-2.12	1.27
ORF:YBL029C-A	user_defined	user_defined_node	1.69	1.24
ORF:YBL064C	user_defined	user_defined_node	7.09	3.08
ORF:YBL108C-A	user_defined	user_defined_node	1.58	2.48
ORF:YBL112C	user_defined	user_defined_node	1.74	3.95
ORF:YBR052C	user_defined	user_defined_node	2.53	1.91
ORF:YBR116C	user_defined	user_defined_node	34.48	1.61
ORF:YBR187W	user_defined	user_defined_node	-2.04	-2.35
ORF:YBR261C	user_defined	user_defined_node	-2.47	-1.38
ORF:YCL021W-A	user_defined	user_defined_node	2.86	-1.16
ORF:YCR099C	user_defined	user_defined_node	1.63	1.39

ORF:YDL025C	user_defined	user_defined_node	3.07	4.15
ORF:YDL124W	user_defined	user_defined_node	2.54	2.61
ORF:YDL187C	user_defined	user_defined_node	-2.05	1.35
ORF:YDL204W	user_defined	user_defined_node	18.67	2.80
ORF:YDR070C	user_defined	user_defined_node	6.50	3.35
ORF:YDR117C	user_defined	user_defined_node	-1.68	1.88
ORF:YDR479C	user_defined	user_defined_node	1.83	-1.21
YGR236C	*SPG1*	Protein required for survival at high temperature during stationary phase; not required for growth on nonfermentable carbon sources; the authentic, non-tagged protein is detected in highly purified mitochondria in high-throughput studies|Spg1p; protein coding	5.34	1.18
ORF:YDR533C	user_defined	user_defined_node	6.99	1.59
YDL223C	*HBT1*	Hbt1p|Substrate of the Hub1p ubiquitin-like protein that localizes to the shmoo tip (mating projection); mutants are defective for mating projection formation, thereby implicating Hbt1p in polarized cell morphogenesis; protein coding	7.30	2.07
ORF:YEL067C	user_defined	user_defined_node	-3.02	1.64
ORF:YER030W	user_defined	user_defined_node	-1.69	1.20
ORF:YFL030W	user_defined	user_defined_node	2.33	1.57
ORF:YFR017C	user_defined	user_defined_node	6.03	1.49
ORF:YGL039W	user_defined	user_defined_node	-2.62	-1.29
ORF:YGL045W	user_defined	user_defined_node	1.83	2.81
ORF:YGL088W	user_defined	user_defined_node	1.88	4.68

ORF:YGL102C	user_defined	user_defined_node	-3.21	-1.77
ORF:YGR043C	user_defined	user_defined_node	10.55	4.48
ORF:YGR086C	user_defined	user_defined_node	4.02	10.06
ORF:YGR111W	user_defined	user_defined_node	2.48	1.24
ORF:YGR130C	user_defined	user_defined_node	2.37	1.84
ORF:YGR151C	user_defined	user_defined_node	-2.03	-1.44
ORF:YGR201C	user_defined	user_defined_node	3.50	1.27
ORF:YHL008C	user_defined	user_defined_node	3.05	1.62
ORF:YHL035C	user_defined	user_defined_node	2.04	2.02
ORF:YHL046C	user_defined	user_defined_node	2.06	2.73
ORF:YHR003C	user_defined	user_defined_node	-1.97	-1.85
ORF:YHR080C	user_defined	user_defined_node	2.00	1.17
ORF:YHR087W	user_defined	user_defined_node	12.35	1.69
ORF:YHR097C	user_defined	user_defined_node	3.32	2.08
ORF:YHR121W	user_defined	user_defined_node	-2.84	-1.61
ORF:YHR138C	user_defined	user_defined_node	3.01	2.45
ORF:YHR149C	user_defined	user_defined_node	-1.97	-1.70
ORF:YIL077C	user_defined	user_defined_node	2.63	2.69
ORF:YIL158W	user_defined	user_defined_node	-2.40	-1.32
ORF:YIL176C	user_defined	user_defined_node	1.86	2.48
ORF:YIR041W	user_defined	user_defined_node	1.75	2.23
ORF:YJL016W	user_defined	user_defined_node	3.15	1.58
ORF:YJL048C	user_defined	user_defined_node	2.09	1.40

ORF:YJL122W	user_defined	user_defined_node	-1.99	-1.42
ORF:YJL142C	user_defined	user_defined_node	4.95	1.77
ORF:YJL144W	user_defined	user_defined_node	5.79	1.44
ORF:YJR039W	user_defined	user_defined_node	2.03	2.84
ORF:YKL027W	user_defined	user_defined_node	-1.61	-1.55
ORF:YKL091C	user_defined	user_defined_node	3.84	1.71
ORF:YKL151C	user_defined	user_defined_node	5.32	3.72
ORF:YKL224C	user_defined	user_defined_node	1.79	2.67
ORF:YKR049C	user_defined	user_defined_node	2.99	1.82
ORF:YLL020C	user_defined	user_defined_node	3.29	2.09
ORF:YLL029W	user_defined	user_defined_node	3.05	2.25
ORF:YLR040C	user_defined	user_defined_node	-1.95	-1.48
ORF:YLR126C	user_defined	user_defined_node	-2.35	1.43
ORF:YLR149C	user_defined	user_defined_node	6.56	1.99
ORF:YLR270W	user_defined	user_defined_node	4.29	3.13
ORF:YLR312C	user_defined	user_defined_node	6.35	19.21
ORF:YLR324W	user_defined	user_defined_node	1.64	1.21
ORF:YLR345W	user_defined	user_defined_node	4.21	2.19
ORF:YLR454W	user_defined	user_defined_node	2.58	1.76
ORF:YML117W	user_defined	user_defined_node	2.16	2.04
ORF:YML125C	user_defined	user_defined_node	-2.09	-2.03
ORF:YMR009W	user_defined	user_defined_node	1.89	4.00
ORF:YMR031C	user_defined	user_defined_node	3.06	1.59

ORF:YMR041C	user_defined	user_defined_node	1.98	7.89
ORF:YMR090W	user_defined	user_defined_node	8.00	7.29
ORF:YMR103C	user_defined	user_defined_node	3.95	4.30
ORF:YMR110C	user_defined	user_defined_node	3.03	1.43
ORF:YMR130W	user_defined	user_defined_node	-1.83	-1.51
ORF:YMR140W	user_defined	user_defined_node	1.90	1.43
ORF:YMR158C-B	user_defined	user_defined_node	3.18	1.13
ORF:YMR173W-A	user_defined	user_defined_node	3.01	1.82
ORF:YMR181C	user_defined	user_defined_node	1.92	3.63
ORF:YMR188C	user_defined	user_defined_node	2.31	1.26
ORF:YMR196W	user_defined	user_defined_node	6.29	5.04
ORF:YMR262W	user_defined	user_defined_node	1.88	1.58
ORF:YMR304C-A	user_defined	user_defined_node	-4.73	-1.99
ORF:YNL080C	user_defined	user_defined_node	1.43	3.26
ORF:YNL115C	user_defined	user_defined_node	3.02	1.90
ORF:YNL134C	user_defined	user_defined_node	4.30	2.43
ORF:YNL156C	user_defined	user_defined_node	2.48	1.21
ORF:YNL195C	user_defined	user_defined_node	11.68	1.19
ORF:YNR047W	user_defined	user_defined_node	2.78	1.23
ORF:YOL019W	user_defined	user_defined_node	-2.33	2.55
YLR093C	*NYV1*	Nyv1p|v-SNARE component of the vacuolar SNARE complex involved in vesicle fusion; inhibits ATP-dependent Ca(2+) transport activity of Pmc1p in the vacuolar membrane; protein coding	1.71	2.72

ORF:YOL083W	user_defined	user_defined_node	2.09	1.43
ORF:YOL087C	user_defined	user_defined_node	2.09	1.61
YMR215W	*GAS3*	Gas3p|Putative 1,3-beta-glucanosyltransferase, has similarity to Gas1p; localizes to the cell wall; protein coding	-2.25	-1.50
ORF:YOR019W	user_defined	user_defined_node	5.11	4.73
ORF:YOR051C	user_defined	user_defined_node	-1.88	-2.16
YDL024C	*DIA3*	Dia3p|Protein of unknown function, involved in invasive and pseudohyphal growth; protein coding	3.91	1.86
